# Genomic and expression profiling reveal molecular heterogeneity of disseminated tumor cells in bone marrow of early breast cancer

**DOI:** 10.1038/s41523-018-0083-5

**Published:** 2018-09-05

**Authors:** Mark Jesus M. Magbanua, Hope S. Rugo, Louai Hauranieh, Ritu Roy, Janet H. Scott, Jen Chieh Lee, Feng Hsiao, Eduardo V. Sosa, Laura van’t Veer, Laura J. Esserman, John W. Park

**Affiliations:** 1 Division of Hematology/Oncology, University of California, San Francisco, San Francisco, CA USA; 2 Helen Diller Family Comprehensive Cancer Center and Computational Biology and Informatics, University of California, San Francisco, San Francisco, CA USA; 3 Department of Laboratory Medicine, University of California, San Francisco, San Francisco, CA USA; 4 Department of Surgery, University of California, San Francisco, San Francisco, CA USA

## Abstract

Detection of disseminated tumor cells (DTCs) in bone marrow is an established negative prognostic factor. We isolated small pools of (~20) EPCAM-positive DTCs from early breast cancer patients for genomic profiling. Genome-wide copy number profiles of DTC pools (*n* = 45) appeared less aberrant than the corresponding primary tumors (PT, *n* = 16). *PIK3CA* mutations were detected in 26% of DTC pools (*n* = 53), none of them were shared with matched PTs. Expression profiling of DTC pools (*n* = 30) confirmed the upregulation of *EPCAM* expression and certain oncogenes (e.g., *MYC* and *CCNE1*), as well as the absence of hematopoietic features. Two expression subtypes were observed: (1) luminal with dual epithelial–mesenchymal properties (high *ESR1* and *VIM/CAV1* expression), and (2) basal-like with proliferative/stem cell-like phenotype (low *ESR1* and high *MKI67/ALDH1A1* expression). We observed high discordance between *ESR1* (40%) and *ERRB2* (43%) expression in DTC pools vs. the clinical ER and HER2 status of the corresponding primary tumors, suggesting plasticity of biomarker status during dissemination to the bone marrow. Comparison of expression profiles of DTC pools with available data from circulating tumor cells (CTCs) of metastatic breast cancer patients revealed gene expression signatures in DTCs that were unique from those of CTCs. For example, *ALDH1A1*, *CAV1*, and *VIM* were upregulated in DTC pools relative to CTCs. Taken together, analysis of pooled DTCs revealed molecular heterogeneity, possible genetic divergence from corresponding primary tumor, and two distinct subpopulations. Validation in larger cohorts is needed to confirm the presence of these molecular subtypes and to evaluate their biological and clinical significance.

## Genetics: two subtypes of disseminated tumor cells in bone marrow

Tumor cells found in the bone marrow of women with early-stage breast cancer constitute molecularly distinct populations that could impact patient outcomes and treatment responses. Mark Jesus Magbanua and coworkers from the University of California, San Francisco, USA, performed genome-wide copy number variation analysis, expression profiling on 64 cancer-related genes and screened for *PIK3CA* mutations in pools of tumor cells isolated from bone marrow aspirates of patients undergoing surgery. These disseminated tumor cells (DTCs) contained fewer genomic abnormalities on average than their corresponding primary tumors, although the DTCs often acquired oncogenic mutations in *PIK3CA*. Expression profiling revealed two distinct subtypes of DTCs, both of which were different from circulating tumor cells found in the bloodstream. Further studies are needed to evaluate the biological and clinical significance of this molecular and genomic heterogeneity in DTCs.

## Introduction

Efforts toward detection and characterization of disseminated tumor cells (DTC) have been actively pursued to shed light on their molecular nature and to evaluate their potential clinical utility as biomarkers.^1–3^ While many studies have now shown that the presence of DTCs is strongly associated with poor patient outcomes,^4–6^ testing for DTCs has not been incorporated into standard clinical practice due to a lack of consensus on methods for detection of these cells.^1,7^ DTC assays have often relied on immunocytochemistry or polymerase chain reaction-based methods to detect the presence of these cells in the bone marrow.^1^ Our group has used EPCAM-based immunomagnetic enrichment and fluorescence-activated cell sorting (IE/FACS) for detection and isolation of circulating tumor cells (CTC) from blood of cancer patients.^8,9^ This method involves an initial IE step using magnetic beads coated with monoclonal antibody to EPCAM, followed by FACS to detect and purify CTCs away from blood cells. Previous studies have demonstrated the robustness of the IE/FACS method for detection and isolation of highly pure CTCs (>90%),^8,9^ and downstream molecular analyses have confirmed the malignant nature of IE/FACS-isolated CTCs.^8–10^

In this study, we applied IE/FACS to detect and isolate pools of EPCAM-expressing DTCs from bone marrow of early breast cancer patients. Pooled cells, along with their matched primary tumors, were subjected to genome-wide copy number analysis and *PIK3CA* mutation screening. We also analyzed the expression of 64 cancer-related genes in DTCs, and compared DTC expression profiles with publicly available CTC gene expression data. Finally, we compared *ESR1* and *ERBB2* expression in DTCs vs. the clinical ER and HER status of corresponding primary tumors.

## Results

### DTCs can be enumerated by IE/FACS

**Fig. 1:**
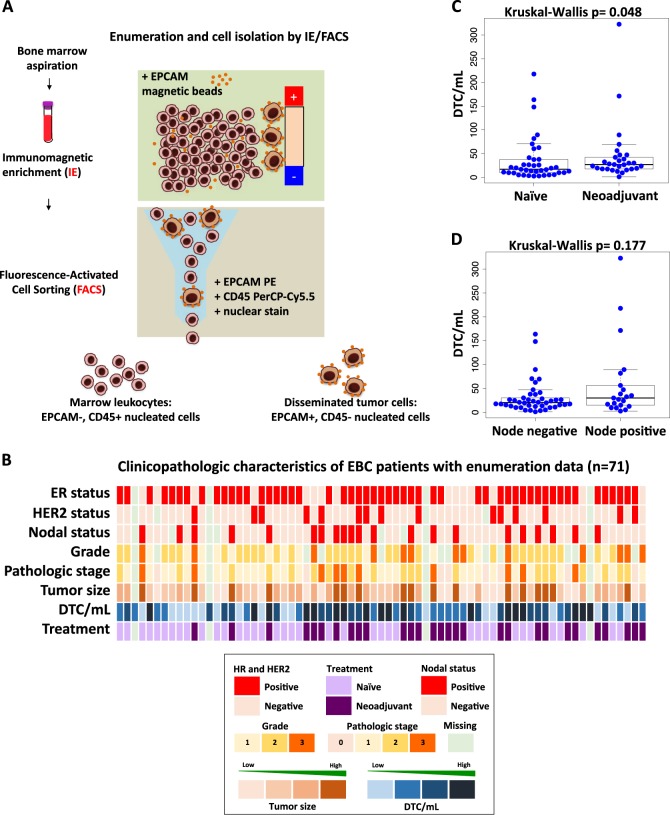
DTCs from bone marrow of early breast cancer patients were enumerated and isolated for downstream molecular profiling. **a** Enumeration and isolation of DTCs using a two-step process involving immunomagnetic enrichment and flow cytometry or fluorescence-activated cell sorting (IE/FACS). **b** Clinical characteristics of 71 patients from whom DTCs were enumerated. Each column represents a patient. **c**–**d** Comparison of DTC/mL between groups based on patient treatment and nodal status (also see Supplementary Fig. 1 for extended analysis)

Bone marrow aspiration was performed in the operating room immediately prior to breast surgery. Samples were then analyzed via IE/FACS assay to detect and enumerate DTCs (Fig. 1a). A total of 71 sequential patients who had detectable DTCs were included in this study (Fig. 1b, Supplementary Table 1). The median age was 51 years old. 30% of patients were node-positive. 73% of patients were ER-positive, and 21% were HER2-positive. 41% received neoadjuvant chemotherapy prior to study entry.

We did not observe any significant correlation between the concentration of DTCs in the bone marrow (DTC/mL) and standard clinical and pathologic variables (Fig. 1c, d, Supplementary Fig. 1). We did observe higher median DTC/mL in patients who received neoadjuvant chemotherapy compared to those who were treatment naive at the time of surgery (Kruskal–Wallis *p* = 0.048) (Fig. 1c). Node-positive patients showed a statistically insignificant trend toward higher median DTC/mL compared to node-negative patients (Kruskal–Wallis *p* = 0.177). The patient with the highest concentration of DTCs (322.8 DTC/mL) also had largest number of axillary lymph node metastases (21 positive nodes, Fig. 1d).

### Molecular characterization of DTCs

After enumeration of DTCs in 4 mLs of bone marrow, the remaining volume was subjected to IE/FACS to isolate pools of DTCs. Downstream molecular analysis of pooled DTCs was performed in overlapping subsets of the 71 patients from whom DTCs were enumerated and isolated (Supplementary Fig. 2A). A flow chart showing the number of patients and samples used for analysis is presented in Supplementary Fig. 2B. DTCs were subjected to genome-wide copy number analysis, *PIK3CA* mutation screening, and gene expression analysis of 64 cancer-related genes. The panel included epithelial and hematopoietic markers, as well as genes involved in proliferation, tumorigenesis, cell death, epithelial-to-mesenchymal transition (EMT), and stem cell-ness (Supplementary Table 2). Results of molecular profiling are described below.

### DTCs appear less genomically aberrant than corresponding primary tumors

**Fig. 2:**
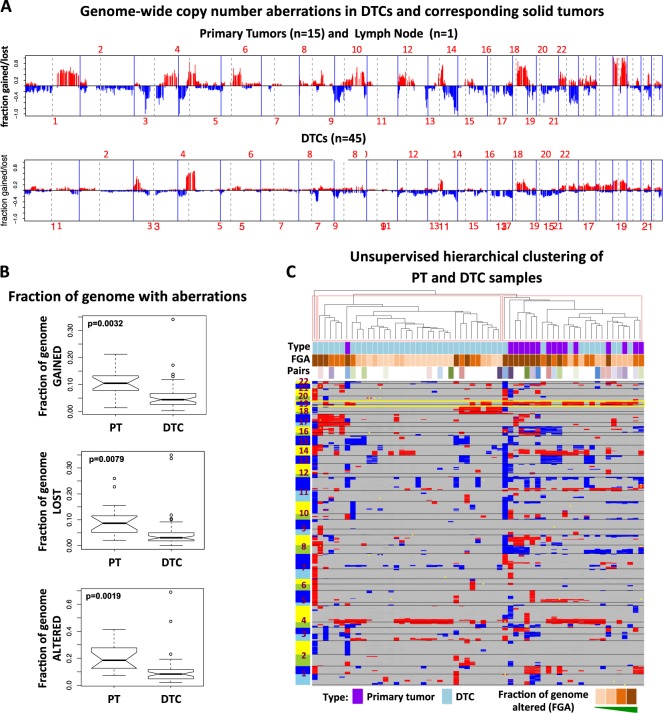
DTCs appear to have less genomic aberrations vs. matched primary tumors. **a** Frequency plot of clone-wise comparisons of archival tumors available from 16 patients (15 primary tumors and 1 lymph node) vs. DTCs (*n* = 45). Red and blue horizontal lines depict frequency of gain and loss, respectively. **b** Comparison of the extent of genomic aberrations in DTCs vs. primary tumors (PT). The *p*-values shown were calculated from fitting a linear model with the log-transformed fraction of genome gained (or lost or altered) as the response variable and sample type as the predictor variable along with patient ID as a covariate. **c** Heatmap based on gain/loss status using Euclidean distance and Ward agglomeration method. Columns represent samples. Chromosomes 1–22 are ordered from bottom to top (rows). Red, blue, and yellow dots represent gain, loss, and amplification, respectively. The yellow box indicates loss of chromosome 19, which is observed predominantly in the right cluster, and appears to drive cluster separation

Pools of DTCs were isolated from 56 of 71 patients in study (79%). Forty-five (80%) of these DTC samples were successfully analyzed by array comparative genomic hybridization (aCGH) (Supplementary Table 3). Genome-wide copy number profiling of matched primary tumors (and one lymph node metastasis) from 16 patients revealed numerous aberrations, including those frequently found in primary breast tumors (e.g., 1q gain, 8p loss, 8q gain, and 16q loss)^11^ (Fig. 2a). DTCs, in general, displayed fewer copy number alterations than the primary tumors (Fig. 2b). Overall, the fraction of genome altered in DTCs was significantly lower compared to that of primary tumors (linear regression (LR) *p* = 0.0019), and so were the fractions of genome gained (LR *p* = 0.0032) and lost (LR *p* = 0.0079). Representative DTC and primary tumor pairs are shown in Supplementary Fig. 3A. Comparative analysis of genomic aberrations between the two groups revealed a significantly higher proportion of primary tumors with 14q12–q21 gain (adjusted *p* = 0.007) and 16q12 loss (adjusted *p* = 0.007) (Supplementary Fig. 3B). Unsupervised hierarchical clustering revealed two major clusters and a singleton DTC sample (Fig. 2c). The left cluster contained mostly DTCs, while the right cluster contained DTC and primary tumor samples. Visual inspection of the heatmap revealed that the loss of chromosome 19, which was observed predominantly in the right cluster, appeared to drive cluster separation. Interestingly, none of the 16 DTC and primary tumor pairs clustered together. Taken together, these results indicate significant divergence of copy number profiles in DTCs as compared with those of the matched primary tumor, and suggest that DTCs generally contain fewer genomic abnormalities than the primary tumor.

### DTCs show frequent *PIK3CA* mutation

**Fig. 3:**
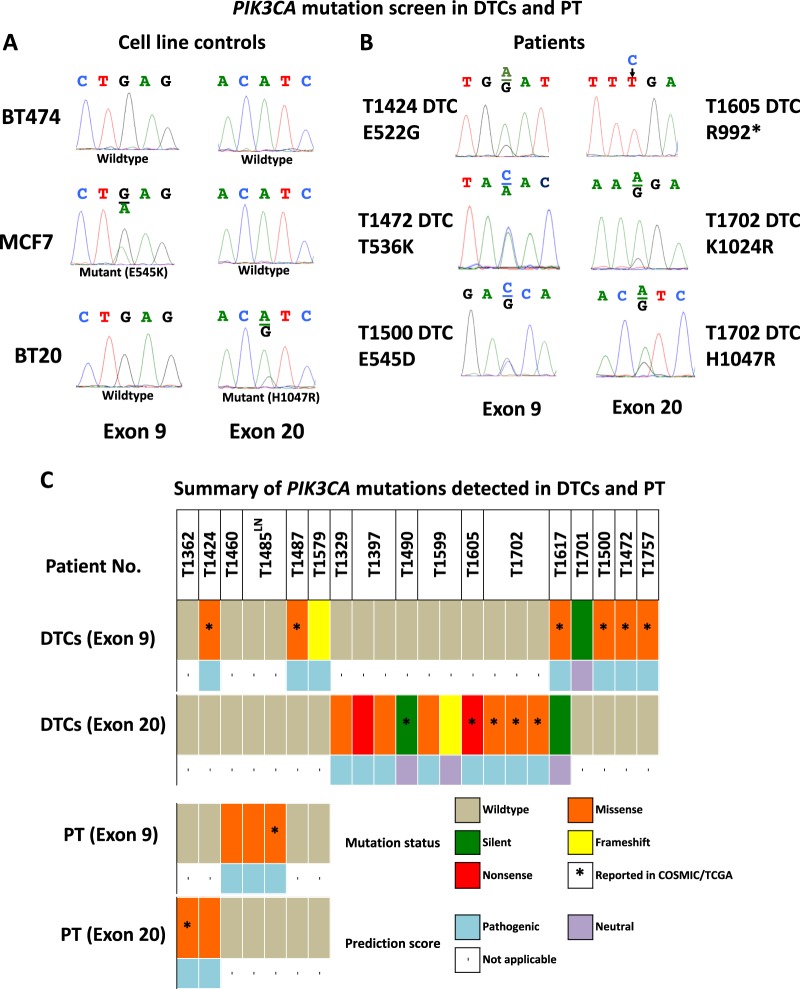
*PIK3CA* mutations can be detected in DTCs. **a** Sanger sequencing traces from cell lines used for assay optimization. Positive controls, MCF7 and BT20 carry mutations E545K (Exon 9) and H1047R (Exon 20), respectively, but not the negative control BT474 cells. **b** Representative Sanger sequencing traces depicting mutations detected in DTCs from five patients. **c** Summary of sequencing data from DTCs, primary tumors, and a lymph node (LN) with at least one mutation detected. The complete list of patients screened for *PIK3CA* mutations and corresponding sequencing results are found in Supplementary Table 4

Next, we screened for *PIK3CA* “hotspot” mutations in 55 of the 56 DTC samples previously analyzed by aCGH. Both Exons 9 and 20 of *PIK3CA*, which includes the mutational hotspots E545 and H1047, respectively, were sequenced (Supplementary Fig. 4). Testing in cell lines with known mutation status revealed specificity of the assay (Fig. 3a). Fifty-three patients had evaluable sequencing data (Supplementary Table 4). Of those, 19 mutations (8 in exon 9 and 11 in exon 20) were detected in DTCs from 14 (26%) of the 53 patients with evaluable sequencing data (Fig. 3b, c). These included 3 silent, 2 frameshift, 2 nonsense, and 12 missense mutations. One of the silent mutations was considered germline, while the rest were not observed in corresponding normal marrow leukocytes. Eleven of the 19 (58%) have been previously reported in the COSMIC and/or TCGA databases, and 15 (80%) were predicted to be pathogenic or probably damaging. In matched archival samples, three primary tumors and the lymph node metastasis (29%) carried *PIK3CA* mutations. No *PIK3CA* mutations were shared between DTCs and matched primary tumors.

### Expression profiling of IE/FACS-isolated DTCs indicate epithelial and malignant origin

**Fig. 4:**
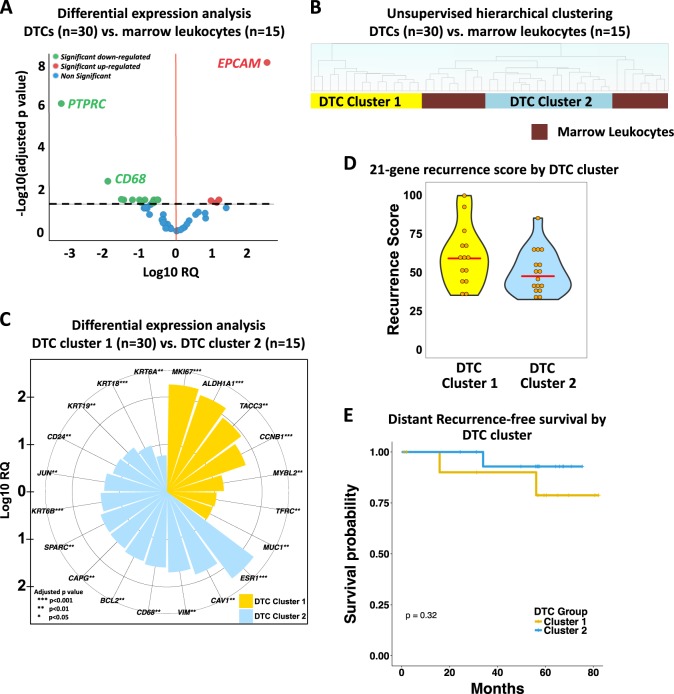
Gene expression analysis reveals two groups of DTCs with distinct expression profiles. **a** Volcano plot showing differentially expressed genes between DTCs and marrow leukocytes. Genes with an adjusted *p*-value < 0.05 (black dashed line) were considered statistically significant. Relative quantification (RQ) is reported in the logarithmic scale (log10 RQ = log10^2-∆∆CT). A Log10 RQ = 1 or −1 means a gene is expressed 10 times or 1/10 as much, respectively, in DTCs relative to marrow leukocyte samples. **b** Unsupervised hierarchical clustering analysis of DTCs (*n* = 30) and CD45-positive marrow leukocytes (*n* = 15) isolated by IE/FACS. **c** A rose plot showing genes upregulated in DTCs in cluster 1 (yellow) and cluster 2 (blue). **d** Violin plot of the 21-gene recurrence scores derived from DTC gene expression data from aQPCR analysis. The red line indicates the median. **e** Kaplan–Meier analysis for recurrence-free survival between patients whose DTCs belong to cluster 1 vs. cluster 2

We isolated pools of DTCs from 35 of the 71 patients in the study (49%), 30 (86%) of whom were successfully subjected to multiplexed Taqman Low-Density Array QPCR (aQPCR) analysis of 64 cancer-related genes (Supplementary Table 5). In parallel, marrow leukocytes from 15 patients were isolated and profiled. Comparison of DTC expression profiles with marrow leukocytes revealed significant upregulation of *EPCAM* and *MUC1*, and downregulation of hematopoietic cell markers, *PTPRC*/*CD45* and *CD68* (Fig. 4a). These results are consistent with our epithelial-based approach for isolation of DTCs. Moreover, oncogenes *CCNE1* and *MYC* were also upregulated in DTCs.

Of the 30 patients whose DTCs were successfully analyzed by aQPCR, 14 had corresponding *PIK3CA* mutation data, 4 (29%) of whom carried non-synonymous *PIK3CA* mutations (Supplementary Fig. 2C). Taken together, the detection of *PIK3CA* mutations and the upregulation of oncogenes *CCNE1* and *MYC* in DTCs suggest malignant phenotype.

### Expression profiling reveals two distinct groups of DTCs

Unsupervised hierarchical clustering analysis revealed two major clusters: a cluster containing only DTCs (DTC cluster 1, *n* = 14), and another cluster which contained both DTCs (DTC cluster 2, *n* = 16) and marrow leukocytes (Fig. 4b). Differential expression analysis revealed that DTCs in each cluster showed significant upregulation of *EPCAM* and downregulation of *PTPRC* relative to marrow leukocytes (Supplementary Fig. 5), confirming their epithelial and non-hematopoietic nature.

Next, we compared the expression profiles of the two DTC clusters. Upregulated genes in DTC cluster 1 relative to cluster 2 (Fig. 4c), included cell proliferation and cancer stem cell-associated genes. For example, upregulated genes included *MKI67*, a proliferation marker; *ALDH1A1*, a cancer stem cell marker;^12^ and *TACC3*, a gene associated with stemness and proliferation.^13,14^ Proliferation-related genes *CCNB1*^15^ and *MYBL2*^16^ were also upregulated, as was *TFRC*, a gene known to be overexpressed in proliferating malignant cells.^17^ *MUC1*, an epithelial and breast cancer marker, was upregulated in this cluster.

Genes upregulated in DTC cluster 2 relative to cluster 1 included *ESR1*, which encodes the estrogen receptor; *CAV1*, a regulator of estrogen-dependent signaling;^18^ and *BCL2*, an anti-apoptotic gene associated with estrogen receptor positivity.^19^ Interestingly, the macrophage marker *CD68* was also upregulated. *CD24*, a gene that is usually downregulated in cancer stem cell marker was upregulated.^20^ Other genes that were upregulated included epithelial cytokeratins, *KRT*18, *KRT*19, *KRT*6A, and *KRT*6B, as well as *VIM1*, a mesenchymal marker.^21^ Other upregulated genes include SPARC (osteonectin), a gene associated with EMT,^22^ and bone metastasis,^23^ and *CAPG*, a putative biomarker for bone metastasis.^24^

The aQPCR assay included a 21-gene signature (Oncotype Dx),^25^ which allows for the calculation of recurrence scores (RS, Supplementary Information). When RS was calculated in our DTC samples (*n* = 30), all samples were classified in the high-risk group (RS ≥ 31) (Fig. 4d). We observed a slight trend toward higher RSs for samples in DTC cluster 1 compared to those in DTC cluster 2, but this was not statistically significant (Mann–Whitney U *p* = 0.1285).

Clinical follow-up revealed that two patients in cluster 1 experienced recurrence (bone and lymph node metastasis, respectively). In cluster 2, one patient developed liver metastasis. With a median follow-up of 58 months, the difference in distant recurrence-free survival between groups was not statistically significant (log-rank *p* = 0.32) (Fig. 4e).

### *ESR1*/ER and *ERBB2*/HER2 status in DTCs vs. matched primary tumors show high discordance

**Fig. 5:**
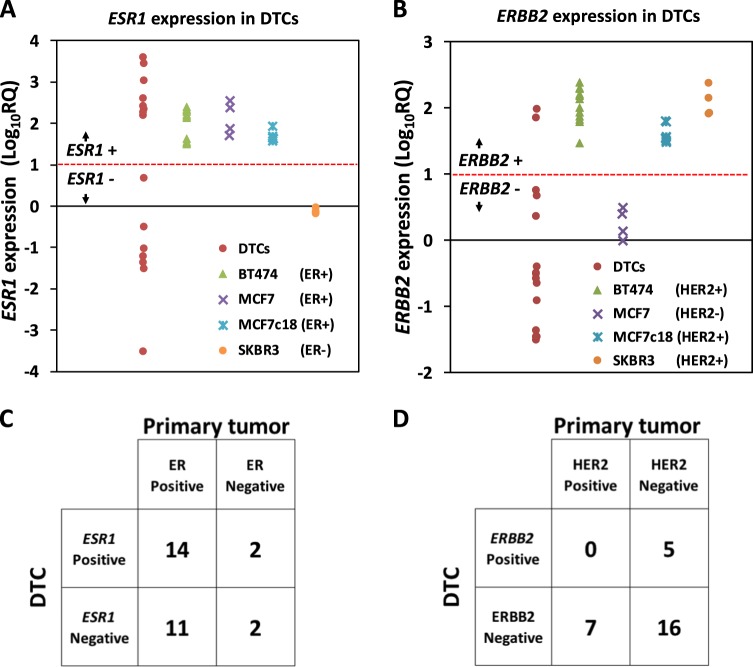
*ESR1*/ER and *ERBB2*/HER2 status in DTCs and matched primary tumors show high discordance. **a** *ESR1* and **b** *ERBB2* expression in DTCs and breast cancer cell lines with known ER and HER2 status, respectively. The red dashed lines indicate the selected cut-off for positivity (Log10 RQ = 1); two-by-two contingency tables showing agreement in **c** *ESR1* and ER status and **d** *ERBB2* and HER2 status in DTCs vs. matched primary tumors

We used gene expression data to assign *ESR1* and *ERBB2* status in 30 DTC samples (Supplementary Information). Detectable expression (Ct < 36) was considered positive. We also calculated relative *ESR1* and *ERBB2* (LogRQ_10_) expression in a subset of DTC samples with corresponding marrow leukocytes (*n* = 15). Using breast cancer cell lines with known ER (Fig. 5a) and HER2 (Fig. 5b) status as references, a Log_10_ RQ ≥ 1 was considered positive. Both approaches (Ct < 36 or Log_10_ RQ ≥ 1) showed high agreement in calls for *ESR1* (93%) and *ERBB2* (87%) expression status.

Based on detectable expression (Ct < 36), 53% (16 of 30) of the DTCs were *ESR1*-positive and 27% (8 of 30) were *ERBB2*-positive (Supplementary Fig. 6A). Interestingly, aCGH analysis did not detect gains or amplification of the *ERBB2* locus in any of DTC samples analyzed (data not shown). Cluster 2 contained a significantly higher proportion of ER-positive DTCs compared to cluster 1 (75 vs. 29%, Chi-squared *p* = 0.0132). In contrast, cluster 1 contained a higher proportion of HER2-positive DTCs compared to cluster 2 (43 vs. 13%), but was not statistically significant (Chi-squared *p* = 0.0695).

Of the 30 DTC samples profiled, matching primary tumor clinical results for ER (*n* = 29) and HER2 (*n* = 28) were available. 86% of the primary tumors were ER-positive, and 25% were HER2-positive. Eleven of the 25 (44%) ER-positive primary tumor samples were associated with *ESR1*-negative DTCs, while 2 of the 4 (50%) ER-negative tumors had ER-positive DTCs (Fig. 5c). All 7 patients with HER2-positive primary tumors had HER2-negative DTCs, while 5 of the 21 (24%) HER2-negative patients had HER2-positive DTCs (Fig. 5d). Overall, the discordance in ER and HER2 status between DTCs and their corresponding primary tumors was high (45%, *κ* = 0.031 and 43%, *κ* = −0.263, respectively).

The proportion of receptor subtypes was significantly different between DTCs and primary tumors (Fisher’s exact *p* = 0.028) (Supplementary Fig. 6B). *ESR1*−*ERBB2*+ and *ESR1*−*ERBB2*− subtypes were more frequent in DTCs, while ER+HER2− and ER−HER2+ subtypes were more frequent in primary tumors. The ER−HER2+ subtype was not represented among the matched primary tumors in this cohort.

### DTC gene expression and neoadjuvant treatment

Fifteen of the 30 patients with DTC expression data had completed neoadjuvant chemotherapy at the time of bone marrow aspiration. Expression profiles of DTCs did not appear to cluster based on whether neoadjuvant treatment was received (Supplementary Fig. 6A). Differential expression analysis between DTCs from treatment-naive vs. neoadjuvant-treated patients did not show significant differentially expressed genes (Supplementary Fig. 6C).

### DTC gene expression profiles appear different from those of CTCs

Next, we compared expression profiles of DTCs with CTC gene expression data recently reported by our group.^9^ In a previous study, CTCs were isolated from blood of metastatic breast cancer patients using the same IE/FACS method, and profiled using the same microfluidic aQPCR platform. The merged data was normalized using the reference genes (*RPS18* and *ACTB*).

**Fig. 6:**
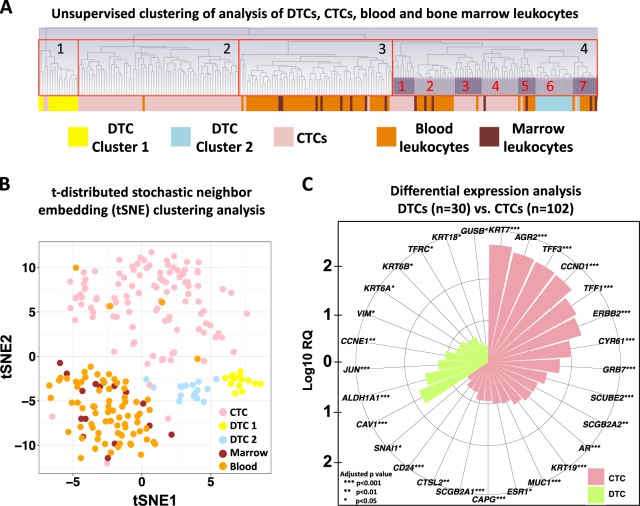
DTCs and CTCs exhibit expression profiles that are unique from each other. **a** Unsupervised hierarchical clustering analysis of CTCs (*n* = 105) and DTCs (*n* = 30) along with matched blood leukocytes (*n* = 76) and marrow leukocytes (*n* = 15). **b** t-SNE analysis to determine clusters based on similarities in gene expression. **c** A rose plot showing genes upregulated in DTCs and CTCs. Genes with an adjusted *p-*value < 0.05 were considered statistically significant. Relative quantification (RQ) is reported in the logarithmic scale (log10 RQ = log10^2-∆∆CT). A Log10 RQ = 1 or −1 means a gene is expressed 10 times or 1/10 as much, respectively

Unsupervised hierarchical clustering of CTCs (*n* = 105), DTCs (*n* = 30), blood (*n* = 76), and bone marrow leukocytes (*n* = 15) revealed four major clusters (Fig. 6a). The first cluster, which formed an entirely separate group, contained identical DTC samples from previously identified cluster 1. The second cluster contained samples that were predominantly CTCs. The third cluster contained a combination of blood and marrow leukocytes. The fourth cluster had seven subclusters, each with mostly the same cell types. Subcluster 6, for example, contained all but one of the DTC samples belonging to the previously identified cluster 2. Two-dimensional t-SNE analysis to identify clusters revealed similar results (Fig. 6b). In general, bone marrow and blood leukocytes clustered together, while CTCs formed a separate cluster. Moreover, DTC samples separated into two groups corresponding to the clusters identified above.

Differential expression analysis revealed that, relative to DTCs, CTCs exhibit upregulation of epithelial (*KRT7*, *KRT19*, *MUC1*) and EMT (*SNAI1*) markers; upregulation of the androgen receptor (*AR*), estrogen-related genes (*ESR1*, *AGR2*, *SCUBE2*, *SCGB2A2*, *SCGB2A1*, *TFF1*, *TFF3*), as well as HER2-related genes (*ERBB2*, *GRB7*) (Fig. 6c). DTCs, on the other hand, displayed upregulation of mesenchymal (*VIM*) and epithelial markers (*KRT6A*, *KRT6B*, *KRT18*), and putative stem cell marker *ALDH1A1*.

## Discussion

Improvements in technologies for rare-cell detection and analysis of limited amount of nucleic acids in the past two decades have facilitated efforts toward isolation and molecular characterization of DTCs.^26–44^ Evidence from early genomic studies of DTCs have provided insights into mechanisms of cancer dissemination and evolution^1^ (also see Supplementary Table 6). We built upon these previous studies by performing copy number, gene expression, and mutation screening in EPCAM-positive DTCs detected in bone marrow of early breast cancer patients. Our results revealed genomic heterogeneity and malignant characteristics in these cells. We also detected two subpopulations of DTCs with differing phenotypic properties.

Approaches for detection of DTCs have frequently relied on immunophenotyping to identify epithelial cells in the bone marrow. Borgen and colleagues^45^ previously outlined a standardized immunocytochemical (ICC) method for detection of micrometastatic cells using pan-cytokeratin (CK) monoclonal antibodies along with morphological criteria to classify cells positive for cytokeratin staining.

In this study, we demonstrated the feasibility of an EPCAM-based approach for detection and isolation of DTCs.^9^ We utilized the cell surface marker EPCAM because it obviated the need for harsh permeabilization (required to access cytoplasmic CK antigens), a procedure that could potentially affect nucleic acid stability.^46^ In addition, EPCAM-based cell capture has been used extensively for CTCs,^47^ and so by isolating DTCs with a parallel approach we could directly compare cancer cells from these two compartments. Similar to CK-based detection, positive EPCAM expression is not a sufficient criterion to consider a cell a cancer cell. Here, we provide molecular evidence that EPCAM-positive cells isolated via IE/FACS from the bone marrow display molecular features that are consistent with a tumor phenotype.

The IE/FACS approach used here was able to recover relatively high numbers of DTCs for analysis. What appears to be a high number of DTCs isolated by IE/FACS in comparison to standard ICC reflects the number of cells analyzed in each assay. The standard ICC assay for DTC detection typically evaluates 4–8 million mononuclear cells per sample. For example, Fehm and colleagues outlined a standardized ICC protocol involving cytospins of Ficoll-separated mononuclear cells onto 2–4 slides (each containing 2 million mononuclear cells).^48^ In contrast, the IE/FACS assay, which routinely utilizes 4 mL of bone marrow, analyzes ~176 million mononuclear cells per sample, a 20-fold or more larger number of cells. On the other hand, the higher sensitivity of IE/FACS is accompanied by lower specificity than standard ICC. In separate larger scale studies, we observed that 68% of bone marrow samples from 584 early breast cancer patients were considered positive for DTCs (Magbanua, in preparation). This is clearly higher than the positivity rate of 30.6% reported in a large pooled analysis of ICC-based studies.^5^

Our genome-wide copy number analysis revealed that DTCs, in general, carried fewer aberrations than the matched primary tumors. These results are consistent with previous studies indicating lesser genomic changes in DTCs vs. primary tumor.^26,29,31,34,37,41,49^ For example, Klein and colleagues isolated DTCs based on CK^26,27^ and EPCAM^28^ expression, and developed a method for genomic profiling of single cells using chromosome comparative genomic hybridization (cCGH) analysis. Single cell studies by Klein et al.^26^ revealed fewer copy number aberrations in DTCs from non-metastatic patients compared to those from patients with metastatic disease. Using the same cCGH method, Schumacher and colleagues^35^ recently reported on copy number analysis in single CK-positive DTCs from patients with non-metastatic esophageal cancer. These investigators also observed that DTCs had fewer aberrations compared to corresponding primary tumors. Detection of genomic aberrancy in DTCs in these studies may have been limited by the low resolution of the cCGH method, which makes it less sensitive in detecting small copy number aberrations.^50^ However, Holcomb and colleagues^49^ used high-resolution array-based CGH analysis in small pools of EPCAM-positive DTCs; they reported that cells isolated from patients with localized prostate cancer had significantly fewer aberrations vs. their corresponding primary tumors.^49^

Alternatively, our results showing relatively lower copy number variations in DTCs may be at least in part attributable to the presence of non-DTCs within the pools analyzed by aCGH. Indeed, one of the major limitations of this study is that cells were analyzed as pools and not as individual cells, thus preventing the examination of copy number profiles at the single cell level. Single cell genomic analysis of CK-positive cells in the bone marrow by Demeulemeester and colleagues^32^ revealed that 53% (10 of 19) of these cells were in fact DTCs, with tumor-specific genomic aberrations consistent with those found in the corresponding primary tumors.^32^ The remaining cells initially classified as tumor cells were found to be “normal” cells (30%) or “aberrant cells of unknown origin” (16%). The authors hypothesized that these non-DTC cells were nonmalignant epithelial cells, hematopoietic lineage cells, or actual tumor cells from an unrelated cancer.

Sequence analysis of *PIK3CA* revealed that about quarter of DTCs carry genetic alterations in this gene. These included novel mutations and those that have been previously documented in breast and other cancers.^11,51^ Some DTCs carried mutations in the *PIK3CA* hot spots, e.g., E545D/G on Exon 9 and H1047R on Exon 20.^11^ Interestingly, the mutations detected in DTCs were not present in the corresponding primary tumors. Other studies have also detected *PIK3CA* mutations in CTCs and DTCs that were not found in their corresponding primary tumors.^52–54^

Consistent with our epithelial-based isolation strategy, we observed upregulation of *EPCAM* and downregulation of *PTPRC* in DTCs. Unsupervised clustering analysis revealed two groups of DTCs with distinct expression profiles. Furthermore, comparisons of expression profiles of DTCs in each cluster with those of marrow leukocytes confirmed upregulation of *EPCAM* and downregulation of *PTPRC*. While *CD68*, a macrophage-specific marker, was overall downregulated in DTCs relative to marrow leukocytes, differential expression analysis between the two DTC clusters showed that DTCs in cluster 2 displayed significantly higher expression levels of *CD68* compared to those in cluster 1. It is possible that DTC samples in cluster 2 had macrophage contamination. However, evidence showing that PTPRC—a pan-leukocyte marker also expressed in macrophages—was not differentially expressed between the two groups does not support this assumption. Lustberg and colleagues^55^ observed atypical CK-positive CTCs in blood of metastatic breast cancer patients that also expressed *CD68*. DTCs in cluster 2 may be enriched for these atypical double positive cells. Furthermore, Adam and colleagues^56^ observed that circulating-associated macrophage-like cells (CAML) physically interact with CTCs in circulation to facilitate tumor dissemination. It is tempting to speculate that *CD68* signals observed in DTC cluster 2 may be coming from CAML cells co-isolated with DTCs. Nonetheless, the upregulation of *CD68* in this DTC cluster warrants further investigation.

Analysis using the 21-gene signature revealed that DTCs in cluster 1 have a numerically higher median RS than those in cluster 2. These results are consistent with gene expression data showing that DTCs in the cluster 1 may have a more aggressive phenotype as they exhibited basal-like (low *ESR1*), proliferative (high *MKI67*), and stem cell-like (high *ALDH1A1*) characteristics. DTCs in cluster 2, in contrast, displayed luminal phenotype (high *ESR1*), low proliferative potential (low *MKI67*), and dual epithelial–mesenchymal characteristics (high *EPCAM* and *VIM* expression), and may represent a less aggressive subtype of DTCs.

Sosa and colleagues have proposed that early during the disease process DTCs transit to niches that either promote tumor dormancy (Ki67-negative DTCs) or proliferative growth (Ki67-positive DTCs).^57^ It is possible that the DTC subtypes observed in this study represent these two different subpopulations of DTCs. For example, the upregulation of *SPARC* (osteonectin), which has been implicated in metastatic dormancy in bone^58^ and downregulation of *MKI67* in DTCs in cluster 2 suggest that these cells may be dormant,^57^ whereas the upregulation of *MKI67* and *CCNB1* in DTCs in cluster 1 suggests a proliferative phenotype.

It has been hypothesized that subpopulations of DTCs possess stem cell-like properties.^59,60^ In this study, we observed that DTCs in cluster 1 not only displayed high expression of the stem cell marker, *ALDH1A1*,^20^ but also of the proliferation marker, *MKI67*, and *TACC3*, a gene that is involved in promoting stemness and cell proliferation.^13,14^ However, functional studies are needed to fully demonstrate stem cell properties in this subset of DTCs.

We compared the expression status of *ESR1* and *ERBB2* in DTCs with the clinical ER and HER2 status of the corresponding primary tumor. We observed high discordance between *ESR1* (40%) and *ERRB2* (43%) status in DTCs vs. the clinical ER and HER2 status of the corresponding primary tumors, suggesting plasticity of biomarker status over the course of the disease.^61^ Previous studies have also shown discordance in ER (28%^62^; 53%^63^) and HER2 (29–42%^64,65^) status between DTCs and matched primary tumors. In contrast, a study using fluorescence in situ hybridization analysis showed that HER2 status in DTCs is highly concordant with that of the corresponding primary tumors.^66^

Comparison of DTC gene expression with previous CTC results showed distinct clustering of DTCs vs. CTCs, and both were clearly distinguishable from normal blood and bone marrow leukocytes. Further analysis indicated upregulation of stem cell marker (*ALDH1A1*) and epithelial–mesenchymal genes (*VIM*, *KRT6A*, *KRT6B*, *KRT18*) in DTCs as compared to CTCs. The observed bi-phenotypic (epithelial and mesenchymal) nature of EPCAM-positive DTCs isolated by IE/FACS may also be due presence of normal bone marrow cells (e.g., mesenchymal stromal cells) in the pools of cells analyzed. Single-cell expression analysis can confirm whether individual cells express both mesenchymal and epithelial markers.

Limitations of the study include the small sample size, and that CTCs were not collected from the same patients, as were the DTCs and primary tumors. Also, we pursued an EPCAM-based isolation strategy to parallel the approach used in many CTC studies^47^; however, we will not capture DTCs lacking EPCAM expression, such as those undergoing EMT.^67^

Previously, we studied the feasibility of IE/FACS for isolation of highly pure CTCs with minimal contaminating hematopoietic cell content^8,9^ to facilitate detailed molecular profiling. In this study, we have extended the use of IE/FACS for direct isolation and in-depth analysis of DTCs. The ability to isolate CTCs and DTCs using this approach provides new opportunities to study these two aspects of cancer metastasis.

## Conclusions

We demonstrate the feasibility of direct isolation and characterization of EPCAM-positive DTCs from early breast cancer patients. Our data revealed molecular heterogeneity among DTCs and suggested possible genetic divergence of these cells from corresponding primary tumor. We also detected two subpopulations of DTCs with distinct expression profiles.

## Methods

### Patient population and samples

Bone marrow samples were collected from newly diagnosed early breast cancer patients (clinical stages I–III) who were recruited to participate in a local study (TIPPING) at the University of California San Francisco (UCSF). The goal of the TIPPING study, which involved 584 early breast cancer patients, was to enumerate DTCs and to evaluate their prognostic value. The results of the TIPPING study will be reported elsewhere. For this present study, we isolated and profiled small pools of DTCs from 71 TIPPING patients with detectable DTCs. Written informed consent was obtained from all participants. The study was conducted under a protocol approved by the UCSF Institutional Review Board.

Bone marrow was collected via a unilateral bone marrow aspiration from the posterior superior iliac crest while patient was under anesthesia immediately prior to surgery. Samples were collected in EDTA-containing tubes and processed within 24 h after bone marrow aspiration (Supplementary Information). Clinical samples were obtained from December 2007 to May 2012. The flow of sample processing is diagrammed in Supplementary Fig. 2.

### DTC enumeration and isolation by IE/FACS

DTCs were enumerated in 4 mL of bone marrow and the remaining volume (median: 7 mLs; range: 2–17 mLs) was used for DTC isolation (Fig. 1a). Iron beads coated with EPCAM monoclonal antibodies were added to bone marrow samples to enrich for EPCAM-positive cells via magnetic capture.^9^ Differentially labeled monoclonal antibodies were added to the enriched sample to distinguish DTCs (nucleated/EPCAM+/CD45−) from bone marrow leukocytes (nucleated/CD45+/EPCAM−) during cells sorting. Small pools of DTCs (~20 cells) were isolated via FACS, and samples were stored −80 °C until further processing.

### Copy number profiling

Genome-wide copy number analysis in DTCs was performed as previously described.^9^ Briefly, whole-genome amplification (WGA) was performed on genomic DNA from small pools of IE/FACS-isolated DTCs. The resulting amplified genomic DNA was used as input for bacterial artificial chromosome aCGH analysis.

### *PIK3CA* mutation analysis

PCR primers were designed to amplify the regions containing the complete Exon 9 and Exon 20 of the *PIK3CA* gene (Supplementary Information). WGA products were used as inputs for PCR. DNA amplicons were sequenced using the Sanger method and the entire exons, which include the *PIK3CA* mutational hotspot regions on amino acid positions 542, 545, and 1047, were screened for mutations. In DTC samples with detectable mutations, amplified whole-genome DNA from corresponding marrow leukocyte was subjected to *PIK3CA* mutation screening as well.

Point mutations identified were subjected to pathogenic analysis to determine phenotypic consequences of amino acid changes using the Functional Analysis through Hidden Markov Models algorithm, following the guidelines in Catalogue of Somatic Mutations in Cancer (COSMIC) for pathogenicity annotation.^68^ Pathogenicity of detected frameshift mutations was predicted using Variant Effect Scoring Tool in the Cancer-Related Analysis of Variants Toolkit web server.^69^

### Expression profiling

The expression of 64 cancer-related genes in DTCs was analyzed as previously described.^9^ Briefly, RT-PCR was performed using a custom Taqman^®^ Low-Density Array (Applied Biosystems) microfluidic card containing the 64 Taqman^®^ gene expression assays printed in triplicate (referred to as aQPCR). To select the optimal gene(s) for normalization, we used the geNorm algorithm within RealTime StatMiner^®^ to calculate the gene stability measure (M) for all six candidate genes (*ACTB*, *GAPDH*, *GUSB*, *RPLP0*, *TFRC*, and *RPS18*). *ACTB* and *RPS18* showed lowest M values indicating most stable expression across all samples, and therefore were chosen as references genes.

### *ESR1*/ER and *ERBB2*/HER status assessment

DTCs with detectable expression (Ct value < 36) of *ESR1* and *ERBB2* were considered positive (Supplementary Information). The clinical ER and HER2 status of corresponding primary tumors were obtained from patients’ medical records.

### Statistical analysis

All statistical analyses were carried out using R/Bioconductor software,^70^ unless otherwise indicated.

#### Copy number analysis

To determine copy number status (gain/loss/normal), the aCGH data was processed using circular binary segmentation, as described previously,^9^ with some modifications. Details of the methods for copy number assessment are discussed in the Supplementary Information.

#### Expression analysis

We used the RealTime StatMiner^®^ version 4.2 to analyze gene expression data. Genes with Cts ≥ 36 were considered unreliable and were flagged as “not detected”. Unsupervised complete linkage hierarchical clustering analyses were performed using Euclidean distance as a similarity measure. Differential expression analysis was performed using a parametric analysis (Limma) for unpaired samples and a paired *t*-test for paired samples. Benjamini–Hochberg method was used to adjust for multiple comparisons. An adjusted *p*-value < 0.05 was considered statistically significant. Relative quantification (RQ) was reported in the logarithmic scale (log_10_RQ = log_10_ 2^-∆∆Ct^). A log_10_RQ = 0 means no differential expression, log_10_RQ = 1 or −1 means a gene is expressed 10 times or 1/10 as much in the test sample relative to the calibrator sample, respectively. Two-dimensional clustering was also performed using t-SNE analysis via the R package Rtnse.^71^ The 21-gene RSs were computed using the R package genefu as described in the Supplementary Information.

## Electronic supplementary material

Supplementary Information

## Data Availability

QPCR and aCGH data has been submitted to the Gene Expression Omnibus (GEO) under the accession numbers GSE112756, GSE112757, and GSE40622.

## Electronic supplementary material

**Supplementary Information** accompanies the paper on the *npj Breast Cancer* website (10.1038/s41523-018-0083-5).
