# Supplementary material for: Genomic and expression profiling reveal molecular heterogeneity of disseminated tumor cells in bone marrow of early breast cancer
Source: NPJ Breast Cancer. 2018 Sep 5;4:31. doi: 10.1038/s41523-018-0083-5 (PMC6125436; doi:10.1038/s41523-018-0083-5)
Supplement: Supplementary file 1 — Supplementary Information [file 41523_2018_83_MOESM1_ESM.pdf]

SUPPLEMENTARY INFORMATION

Magbanua at al. Genomic and expression profiling reveal molecular heterogeneity of disseminated tumor cells in early breast cancer

Workflow of sample processing. Bone marrow aspirates were drawn into EDTA tubes for DTC enumeration. Additional volume of bone marrow was drawn into tubes containing EDTA for DTC isolation.

DTC enumeration. Samples were immediately processed upon receipt. Enumeration of DTCs in ~4 mL of bone marrow was performed using a previously described protocol [1], but with some modifications. Bone marrow sample was diluted with one-half volume of casein buffer and transferred to 7 mL glass tube. The sample was then enriched for epithelial cells by adding 96 µL of anti-EpCAM (MJ37)-coated iron nanoparticles. A second monoclonal antibody against EpCAM (EBA-1) conjugated to phycoerythrin was also added to the sample for subsequent flow cytometric detection. The tube was subjected to a magnetic field for 15 min, and the unbound cells (supernatant) were aspirated. The sample was removed from the magnet and the remaining cells remaining were resuspended in 2 mL of casein buffer. The resuspended cells were transferred to a 12 x 75 polystyrene tube and subjected to second round of magnetic separation for 5 min. The supernatant was aspirated and the cells were resuspended in 150 µL of 1x phosphate buffered saline (PBS). A 20-µL solution containing equal volumes of anti-CD45 (2D1) PerCP-Cy5.5 and a nucleic acid dye, Thioflavin (BD Biosciences), were added. The sample was incubated in the dark for 15 min, and then 1x PBS was added prior to flow cytometry. All reagents and antibodies were obtained from BD Biosciences.

The optimized FACS gating strategy shown in **Supplementary Figure 7** was used for isolation of DTCs. In addition to forward and side scatters, the fluorescence signal intensities from the following stains: (1) EPCAM (EBA-1) mAb conjugated to phycoerythrin (EPCAM-PE), (2) a nucleic acid dye, thioflavin, and (3) a leukocyte-specific CD45 (2D1) mAb conjugated to peridinin-chlorophyll-protein-Cy5.5 (CD45-PerCPCy5.5) were assessed for each event. Forward and side scatters were used for preliminary identification of cells

(P1 gate) and to exclude debris. P2 was used to gate for nucleated (nucleic acid dye+) cells and the next two gates were used to select for EPCAM+/CD45- (P3 gate) and EPCAM+/nucleated cells (P4 gate). DTCs must be present within gates P1–P4, and were defined as EPCAM+/CD45-/nucleated cells. P5 was used to gate for marrow leukocytes (non-tumor controls), which are defined as CD45+/EPCAM-/nucleated cells.

DTC isolation. DTCs were isolated within 24 hours after bone marrow aspiration using the same procedure. Cells were sorted into reaction tubes containing appropriate solution for DNA or RNA profiling and then stored in -80°C until further processing (**Supplementary Figure 2A**). In a subset of samples, replicate pools of DTCs from the same enriched sample were collected for parallel DNA and RNA profiling.

Quality control of samples. Quality control of DNA and RNA data was performed as previously described [2]. Performance of each sample against different quality control steps is shown in **Supplementary Table 2-4** and **Supplementary Figure 2B**.

Copy number analysis. The sample median absolute deviation (MAD) was calculated to estimate noise in individual aCGH data. Clones with segmented value equal to the median segment value of autosomal clones were considered to have a normal copy number. Copy number status of clones with segmented value that is one sample MAD higher than normal were considered as “gain”, while those that had one sample MAD lower than normal were considered as “loss”. A clone was considered to be amplified if its log2 ratio value was 4 sample MAD above the segmented value and was greater than the "normal" segment value by at least 0.75. To estimate the amount of the fraction of the genome altered (FGA), each clone was assigned a genomic distance equal to the sum of one half the distance between its center and that of its neighboring clones. The genomic distances of clones that were gained or lost were added to calculate the FGA. The frequency of gains or losses between primary tumors and DTCs was compared in a clone-wise manner. For each clone, a linear model was fit with the segmented value as the response variable and sample type as the predictor variable along with patient ID as a covariate. Q-values were then

computed to correct for multiple testing. To compare the extent of genomic aberrations between primary tumors and DTCs, a linear model was fit with the log-transformed fraction of genome altered (or gained or lost) as the response variable and sample type as the predictor variable along with patient ID as a covariate.

Mutation analysis of *PIK3CA*. PCR primers were designed to amplify the regions containing the complete exon 9 and exon 20 of the *PIK3CA* gene (**Supplementary Figure 4**). To increase specificity and prevent non-target amplification of the *PIK3CA* pseudogene on chromosome 22, a thymine nucleotide in the reverse primer for exon 9 was replaced with a locked nucleic acid (LNA) residue [3]. The PCR primer sequences were also designed to contain flanking universal primers M13F(-21) and M13R for sequencing.

The PCR conditions were optimized using breast cancer cell lines as controls: MCF7 and BT20, which carry the hotspot mutations E545K in exon 9 and H1047R in exon 20, respectively, as well as BT474, which carry the wildtype sequences for both exons.

The optimized PCR conditions were as follows: (1) initial denaturation step at 95 °C for 5 minutes, (2) followed by 34 cycles of denaturation at 95 °C for 30 seconds, primer annealing at 60 °C for 60 seconds, and elongation at 72 ° for 1 minute, and (3) a final elongation step at 72 °C for 5 minutes.

The PCR products were run on 2.5% agarose gel to confirm amplification. The amplicons were then sequenced using the Sanger method via a commercial sequencing facility. The sequencing traces were processed and analyzed using the ApE software. Both forward and reverse sequencing reactions were performed to confirm results.

***ESR1* and *ERBB2* status in DTCs**. To determine whether the assignment of *ESR1* and *ERBB2* status in DTCs based on the criteria: detection (Ct<36) or no detection (Ct≥36) was biologically consistent, we calculated the Log_{10}RQs for *ESR1* and *ERBB2* in 15 of the 30 DTC samples with paired marrow leukocytes, and compared them with those of breast cancer cell lines with known ER and HER2 status. Using expression in cell lines as references, $Log_{10}RQ>1$ was chosen as a cut-off for positivity (see **Figure 5A and B** in the main

text). Cell lines studied included: BT474 (ER-positive, HER2-positive); MCF7 (ER-positive, HER2-negative); MCF7clone18 (ER-positive, HER2-positive [stably transformed to over-express *ERBB2*]); and SKBR3 (ER-negative; HER2-positive). $Log_{10}RQ$ values for cell lines were calculated using leukocytes from healthy donors (n=5) as a calibrator group. Comparison of the *ESR1* and *ERBB2* calls using these two approaches (based on Ct or $Log_{10}RQ$) revealed good concordance (see main text).

Oncotype Dx score. We designed our aQPCR platform to include all the genes in the 21-gene recurrence score assay (Oncotype Dx, Genomic Health, Inc.) [4]. For data normalization, delta Cts were calculated by subtracting the mean Ct of the reference genes (see **Supplementary Table 2**) to the Cts of each of the remaining 16 genes in the signature. The delta Cts were converted to negative delta Ct so that higher values correspond to higher levels of expression. The oncotypedx function in the R package genefu (Computation of Gene Expression-Based Signatures in Breast Cancer) was then used to calculate recurrence scores (RS), which estimate the risk of distant recurrence [5].

In a study by Dowsett and colleagues [5], the nine-year distant recurrence rates in low (RS < 18), intermediate (RS = 18 to 30), and high RS (RS≥31) groups were 4%, 12%, and 25%, respectively, in node-negative patients; while in node-positive patients the distant recurrence rates were and 17%, 28%, and 49%, respectively.

REFERENCES:

1 Campbell, M. J., Scott, J., Maecker, H. T., Park, J. W. & Esserman, L. J. Immune dysfunction and micrometastases in women with breast cancer. *Breast Cancer Res Treat* **91**, 163-171, doi:10.1007/s10549-004-7048-0 (2005).

2 Magbanua, M. J. M. *et al.* Expanded Genomic Profiling of Circulating Tumor Cells in Metastatic Breast Cancer Patients to Assess Biomarker Status and Biology Over Time (CALGB 40502 and CALGB 40503, Alliance). *Clin Cancer Res* **24**, 1486-1499, doi:10.1158/1078-0432.CCR-17-2312 (2018).

3 Ang, D. *et al.* Novel method for PIK3CA mutation analysis: locked nucleic acid--PCR sequencing. *J Mol Diagn* **15**, 312-318, doi:10.1016/j.jmoldx.2012.12.005 (2013).

4 Paik, S. *et al.* A multigene assay to predict recurrence of tamoxifen-treated, node-negative breast cancer. *N Engl J Med* **351**, 2817-2826, doi:NEJMoa041588 [pii] 10.1056/NEJMoa041588 (2004).

5 Dowsett, M. *et al.* Prediction of risk of distant recurrence using the 21-gene recurrence score in node-negative and node-positive postmenopausal patients with breast cancer treated with anastrozole or tamoxifen: a TransATAC study. *J Clin Oncol* **28**, 1829-1834, doi:10.1200/JCO.2009.24.4798 (2010).

Supplementary Table 1. Clinicopathologic characteristics of 71 early breast cancer patients from whom DTCs were enumerated.

**Variables**		
	Median	Range
Age	51.2	26-73
	1.4	0
	N	%
ER status		
positive	52	73
negative	15	21
no data	4	6
HER2 status		
positive	15	21
negative	50	70
no data	6	8
Nodal status		
positive	21	30
negative	42	59
no data	8	11
Grade		
1	12	17
2	32	45
3	12	17
no data	15	21
Pathological stage		
0	8	12
1	29	43
2	20	30
3	10	15
no data	4	6
Treatment		
Naïve	29	55
Neodjuvant	29	41
no data	3	4

Supplementary Table 2. List of 64 genes, which include the 21-gene recurrence signature (Oncotype DX), selected for Taqman™ Low Density Array (TLDA) aQPCR analysis of disseminated tumor cells.

Gene Symbol[a]	Assay ID	Gene Name	a.k.a in 21-gene recurrence score test	Group in 21-gene assay
ABCB1	Hs00184500_m1	ATP-binding cassette, sub-family B (MDR/TAP), member 1		
ACTB	Hs99999903_m1	actin, beta	Beta-actin	Reference
AGR2	Hs00180702_m1	anterior gradient homolog 2 (Xenopus laevis)		
ALDH1A1	Hs00946916_m1	aldehyde dehydrogenase 1 family, member A1		
AR	Hs00171172_m1	androgen receptor		
AURKA	Hs00269212_m1	aurora kinase A	STK15	Proliferation
BAG1	Hs00185390_m1	BCL2-associated athanogene	BAG1	Other
BCL2	Hs00608023_m1	B-cell CLL/lymphoma 2	Bcl2	Estrogen
BIRC5	Hs00978503_m1	baculoviral IAP repeat-containing 5	Survivin	Proliferation
CAPG	Hs00156249_m1	capping protein (actin filament), gelsolin-like		
CAV1	Hs00971716_m1	caveolin 1, caveolae protein, 22kDa		
CCNB1	Hs99999188_m1	cyclin B1	Cyclin B1	Proliferation
CCND1	Hs00765553_m1	cyclin D1		
CCNE1	Hs01026536_m1	cyclin E1		
CD24	Hs02379687_s1	CD24 molecule		
CD44	Hs01075861_m1	CD44 molecule		
CD68	Hs00154355_m1	CD68 molecule	CD68	Other
CEACAM1	Hs00236077_m1	carcinoembryonic antigen-related cell adhesion molecule 1 (biliary glycoprotein)		
CTSL2; CTSV	Hs00952036_m1	cathepsin L2	Cathepsin L2	Invasion
CYR61	Hs00155479_m1	cysteine-rich, angiogenic inducer, 61		
EGFR	Hs01076091_m1	epidermal growth factor receptor (erythroblastic leukemia viral (v-erb-b) oncogene homolog, avian)		
EPCAM	Hs00158980_m1	epithelial cell adhesion molecule		
ERBB2	Hs01001580_m1	v-erb-b2 erythroblastic leukemia viral oncogene homolog 2, neuro/glioblastoma derived oncogene homolog (avian)	HER2	HER2
ESR1	Hs01046818_m1	estrogen receptor 1	ER	Estrogen
FOLH1	Hs00379515_m1	folate hydrolase (prostate-specific membrane antigen) 1		
FOXC2	Hs00270951_s1	forkhead box C2 (MFH-1, mesenchyme forkhead 1)		
GAPDH	Hs99999905_m1	glyceraldehyde-3-phosphate dehydrogenase	GAPDH	Reference
GRB7	Hs00180450_m1	growth factor receptor-bound protein 7	GRB7	HER2
GSTM1	Hs02341469_m1	glutathione S-transferase mu 1	GSTM1	Other
GUSB	Hs99999908_m1	glucuronidase, beta	GUS	Reference
IGF1R	Hs99999020_m1	insulin-like growth factor 1 receptor		Reference
JUN	Hs99999141_s1	jun oncogene		
KLK3	Hs03063374_m1	kallikrein-related peptidase 3		
KRT18	Hs01653110_s1	keratin 18		
KRT19	Hs00761767_s1	keratin 19		
KRT5	Hs00361185_m1	keratin 5		
KRT6A	Hs01699178_g1	keratin 6A		
KRT6B	Hs00745492_s1	keratin 6B		
KRT7	Hs00559840_m1	keratin 7		
MDM2	Hs01066930_m1	Mdm2 p53 binding protein homolog (mouse)		
MKI67	Hs01032443_m1	antigen identified by monoclonal antibody Ki-67	KI-67	Proliferation
MMP11	Hs00171829_m1	matrix metallopeptidase 11 (stromelysin 3)	Stromelysin 3	Invasion
MMP9	Hs00234579_m1	matrix metallopeptidase 9 (gelatinase B, 92kDa gelatinase, 92kDa type IV collagenase)		
MUC1	Hs00159357_m1	mucin 1, cell surface associated		
MYBL2	Hs00942543_m1	v-myb myeloblastosis viral oncogene homolog (avian)-like 2	MYBL2	Proliferation
MYC	Hs00153408_m1	v-myc myelocytomatosis viral oncogene homolog (avian)		
PGR	Hs01556702_m1	progesterone receptor	PR	Estrogen
PTGS2	Hs00153133_m1	prostaglandin-endoperoxide synthase 2 (prostaglandin G/H synthase and cyclooxygenase)		
PTPRC	Hs00894734_m1	protein tyrosine phosphatase, receptor type, C		
RPLP0	Hs99999902_m1	ribosomal protein, large, P0	RPLPO	Reference
RPS18	Hs02387368_g1	ribosomal protein S18		
SCGB2A1	Hs00267180_m1	secretoglobin, family 2A, member 1		
SCGB2A2	Hs00935948_m1	secretoglobin, family 2A, member 2		
SCUBE2	Hs00221277_m1	signal peptide, CUB domain, EGF-like 2	SCUBE2	Estrogen
SNAI1	Hs00195591_m1	snail homolog 1 (Drosophila)		
SPARC	Hs00234160_m1	secreted protein, acidic, cysteine-rich (osteonectin)		
TACC3	Hs00170751_m1	transforming, acidic coiled-coil containing protein 3		
TERT	Hs00972656_m1	telomerase reverse transcriptase		
TFF1	Hs00907239_m1	trefoil factor 1		
TFF3	Hs00173625_m1	trefoil factor 3 (intestinal)		
TFRC	Hs00951083_m1	transferrin receptor (p90, CD71)	TRFC	Reference
TUBB3	Hs00964965_m1	tubulin, beta 3		

[a] Genes in bold part of the 21-gene recurrence score assay (Oncotype Dx)

Supplementary Table 3. List of patients and samples for genome-wide copy number profiling and sample performance at each step of quality control.

Patient ID	DTC/ml	Volume of bm for IE/FACS	No. of DTCs sorted for WGA	Matched sample type	Passed WGA QC (yield >5ug)	Passed WGA QC (PCR test)	Passed ACGH QC
T1249	21	n.d.	250	n.a.	yes	n.p.	yes
T1260	38	n.d.	173	PT	yes	n.p.	yes
T1294	19.4	n.d.	103	n.a.	yes	n.p.	yes
T1300	2.7	n.d.	200	n.a.	yes	n.p.	yes
T1310	148.3	n.d.	117	PT	yes	n.p.	yes
T1317	16.3	n.d.	28	PT	yes	n.p.	yes
T1320	15.5	n.d.	400	PT	yes	n.p.	yes
T1329	2.9	5	204	n.a.	yes	yes	yes
T1335	9.2	14	80	n.a.	yes	n.p.	yes
T1339	5.2	15	93	PT	yes	n.p.	yes
T1340	9.6	8	75	n.a.	yes	n.p.	yes
T1342	4.5	7	13	n.a.	yes	yes	yes
T1344	36.8	8	100	n.a.	yes	n.p.	yes
T1347	5.7	16	50	n.a.	yes	n.p.	yes
T1362	26.5	n.d.	50	PT	yes	n.p.	yes
T1382	27.1	n.d.	38	n.a.	yes	n.p.	yes
T1389	4.2	n.d.	50	n.a.	yes	n.p.	no
T1397	20	n.d.	14	n.a.	yes	n.p.	yes
T1399	163.7	n.d.	20	n.a.	yes	n.p.	yes
T1416	9.9	7	50	n.a.	yes	n.p.	yes
T1424	89.5	n.d.	20	PT	yes	n.p.	yes
T1425	24.8	n.d.	20	PT	yes	n.p.	yes
T1427	12.3	n.d.	20	n.a.	yes	n.p.	yes
T1448	9.9	9	20	PT	yes	yes	yes
T1460	14.5	n.d.	20	PT	yes	n.p.	yes
T1472	47.3	14	20	n.a.	yes	n.p.	no
T1478	171.4	4	20	n.a.	yes	n.p.	yes
T1485	24.1	15	20	LN	yes	yes	yes
T1487	26	n.d.	20	PT	yes	yes	yes
T1490	47.4	n.d.	20	n.a.	yes	yes	yes
T1500	81.8	4	20	n.a.	yes	no	n.a.
T1505	30	6	19	n.a.	yes	yes	no
T1553	35.1	7	20	n.a.	yes	yes	yes
T1579	69.7	n.d.	20	PT	yes	yes	yes
T1580	17	n.d.	6	n.a.	Yes	no	n.a.
T1586	60.3	5	20	n.a.	yes	yes	yes
T1599	89.6	6	20	PT	yes	yes	yes
T1605	21.1	7	20	PT	yes	yes	yes
T1617	42.3	5	18	n.a.	yes	yes	yes
T1661	32.6	5	20	PT	yes	yes	yes
T1689	19.6	6	20	n.a.	yes	no	n.a.
T1701	2.2	6	20	n.a.	yes	no	n.a.
T1702	19	6	20	n.a.	yes	no	n.a.
T1707	17.8	5	20	n.a.	yes	yes	yes
T1714	15	5	20	n.a.	yes	yes	yes
T1717	17.5	5	20	n.a.	yes	yes	yes
T1742	41.5	5	20	n.a.	yes	yes	yes
T1744	26.6	3	20	n.a.	yes	yes	yes
T1757	23.9	12	20	n.a.	yes	yes	yes
T1777	56.3	15	20	n.a.	yes	no	n.a.
T1796	37.6	10	20	n.a.	no	no	n.a.
T1798	38.5	9	20	n.a.	yes	yes	yes
T1802	23.4	7	20	n.a.	yes	yes	yes
T1810	5.2	8	20	n.a.	yes	yes	yes
T1822	30.2	8	6	n.a.	yes	yes	no
T1823	322.8	8	20	n.a.	yes	yes	yes

Abbreviations:

WGA: whole genome amplification; IE/FACS: immunomagnetic enrichment and fluorescence activated cells sorting; ACGH: array comparative genomic hybridization; bm: bone marrow; QC: quality control; n.d.: no data; n.a.: not applicable; n.p: not performed; PT: primary tumor; LN: lymph node

Supplementary Table 4. List of patients and samples for *PIK3CA* mutation analysis and sample performance at each step of quality control.

Patient ID	Exon 9					Exon 20				
	Primary tumor	FATHMM Prediction (score)	Disseminated tumor cells	FATHMM Prediction (score)	Mutation present in marrow leukocytes	Primary tumor	FATHMM Prediction (score)	Disseminated tumor cells	FATHMM Prediction (score)	Mutation present in marrow leukocytes
T1249	WT		WT			WT		WT		
T1260	WT		WT			WT		WT		
T1310	WT		WT			WT		WT		
T1317	WT		WT			WT		WT		
T1320	WT		WT			WT		WT		
T1339	WT		WT			WT		WT		
T1362	WT		WT			**H1047R**	Pathogenic (0.96)	WT		
T1424	WT		**E522G**	Pathogenic (0.91)	No	A1046G	Pathogenic (0.96)	WT		
T1425	WT		WT			WT		WT		
T1448	WT		WT			WT		WT		
T1460	I543T	Pathogenic (0.96)	WT			WT		WT		
T1485[b]	**N526K**[a]<br>**Q546R**	Pathogenic (0.82)<br>Pathogenic (0.97)	WT			WT		WT		
T1487	WT		**D520N**	Pathogenic (0.99)	No	WT		WT		
T1579	WT		Q546GfsX32	Pathogenic[c]	No	WT		WT		
T1294			WT					WT		
T1300			WT					WT		
T1329			WT					D1018V	Pathogenic (0.96)	No
T1335			WT					WT		
T1340			WT					WT		
T1342			WT					WT		
T1344			WT					WT		
T1347			WT					WT		
T1382			WT					WT		
T1389			WT					WT		
T1397			WT					Q1014*stop<br>Q1033L	Pathogenic (1.00)<br>Pathogenic (0.99)	No
T1399			WT					WT		
T1416			WT					WT		
T1427			WT					WT		
T1472			**T536K**	Pathogenic (0.80)	No			WT		
T1478			WT					WT		
T1490			WT					**T1025T**	Neutral (0.30)	Yes
T1500			**E545D**	Pathogenic (0.80)	No			WT		
T1505			WT					WT		
T1553			WT					WT		
T1580			WT					WT		
T1586			WT					WT		
T1599			WT					T1061R<br>Q993PfsX25	Pathogenic (0.96)<br>Neutral[c]	No
T1605			WT					**R992*stop**	Pathogenic (0.98)	No
T1617			**L531P**	Pathogenic (0.80)	No			A1046A		
T1661			WT					WT		
T1698			WT					failed		
T1701			L531L	Neutral (0.29)	No			WT		
T1702			WT					**H1047R**<br>**L1013P**<br>**K1024R**	Pathogenic (0.96)<br>Pathogenic (1.00)<br>Pathogenic (1.00)	Not available
T1707			WT					WT		
T1714			WT					WT		
T1717			WT					WT		
T1742			WT					WT		
T1744			WT					WT		
T1757			**E545G**	Pathogenic (0.97)	No			WT		
T1777			WT					WT		
T1796			WT					WT		
T1798			WT					WT		
T1802			WT					failed		
T1822			failed					failed		
T1823			failed					failed		

Supplementary Table 5. List of patients and samples for gene expression analysis and sample performance at each step of quality control.

Patient ID	DTC /mL	Volume of bm for IE/FACS	No. of DTCs sorted for gene expression	Matched sample type	Passed ≥ 10 Cells QC	Passed IGA QC	Passed TLDA QC
T1579	69.7	8.5	20	CD45	Yes	Yes	Yes
T1586	60.3	5	20	CD45	Yes	Yes	Yes
T1599	89.6	6	20	CD45	Yes	Yes	Yes
T1605	21.1	7	20	n.a.	Yes	Yes	Yes
T1617	42.3	8	5	n.a.	No	n.a.	n.a.
T1689	19.6	6	20	n.a.	Yes	Yes	Yes
T1702	19	6	20	n.a.	Yes	Yes	Yes
T1707	17.8	4	20	n.a.	Yes	Yes	Yes
T1710	17.6	6	20	n.a.	Yes	Yes	Yes
T1714	15	5	20	n.a.	Yes	No	n.a.
T1717	17.5	5	20	n.a.	Yes	Yes	Yes
T1742	41.5	12	20	CD45	Yes	Yes	Yes
T1744	26.6	3	20	n.a.	Yes	Yes	Yes
T1755	10	5	20	CD45	Yes	Yes	Yes
T1756	3.6	2	20	n.a.	Yes	No	n.a.
T1757	23.9	14	20	n.a.	Yes	Yes	No
T1777	56.3	15	20	CD45	Yes	Yes	Yes
T1780	70.5	8	20	n.a.	Yes	Yes	Yes
T1794	217.8	7	20	n.a.	Yes	Yes	Yes
T1796	37.6	10	16	n.a.	Yes	Yes	Yes
T1798	38.5	9	20	n.a.	Yes	Yes	Yes
T1802	23.4	7	20	CD45	Yes	Yes	Yes
T1810	5.2	8	20	CD45	Yes	Yes	Yes
T1818	13.7	17	20	CD45	Yes	Yes	Yes
T1822	30.2	5.5	20	n.a.	Yes	Yes	Yes
T1823	322.8	8	20	CD45	Yes	Yes	Yes
T1824	62.7	7.5	20	CD45	Yes	Yes	Yes
T1830	42.6	4	20	CD45	Yes	Yes	Yes
T1838	12.6	9	10	n.a.	Yes	Yes	Yes
T1841	28.4	5	20	n.a.	Yes	Yes	Yes
T1850	11	15	20	CD45	Yes	Yes	Yes
T1852	13.1	5	20	CD45	Yes	Yes	Yes
T1856	1.2	4	20	n.a.	Yes	Yes	Yes
T1859	26.6	4	20	CD45	Yes	Yes	Yes
T1866	15.5	4	12	n.a.	Yes	No	n.a.

Abbreviations:

IE/FACS: immunomagnetic enrichment and fluorescence activated cells sorting; bm: bone marrow; QC: quality control; IGA: individual gene assay; TLDA: Taqman low density array; n.a.: not applicable; CD45: (CD45+) marrow cells.

Supplementary Table 6. List of scientific literature from 1999 to 2018 on detection and molecular characterization of disseminated tumor cells (DTCs).

Study	Type of cancer	Stage	Definition of DTCs	Input for molecular analysis	Molecular assay utilized	Type of molecular analysis
Klein et al, 1999 PNAS	Cancer of unknown primary	Metastatic	Cytokeratin-positive	Isolated single cells	cCGH, Microsatellite analysis, PCR-RFLP, Sanger sequencing	Copy number, LOH, Mutation screen
Klein et al, 2002 Lancet	Breast, prostate, and gastro-intestinal	Non-metastatic and metastatic	Cytokeratin-positive	Isolated single cells	cCGH, Single-stranded conformational polymorphism analysis, Sanger sequencing	Copy number, Mutation screening
Klein et al, 2002 Nat Biotechnol	Cervival, prostate, and breast	Non-metastatic and metastatic	EPCAM-positive	Isolated single cells	cCGH, Dot-blot hybridization	Gene expression, Copy number
Schmidt-Kittler et al, 2003 PNAS	Breast	Non-metastatic and metastatic	Cytokeratin-positive	Isolated single cells	cCGH, Microsatellite analysis	Copy number, LOH
Kraus et al, 2003 Genes Chromosomes Cancer	Prostate	Non-metastatic	n.a.	Bulk cultured cells	cCGH, aCGH, M-FISH	Copy number
Gangnus et al, 2004 Clin Cancer Res	Breast	Non-metastatic	Cytokeratin-positive	Isolated single cultured cells	cCGH	Copy number
Schardt et al, 2005 Cancer Cell	Breast	Non-metastatic and metastatic	Cytokeratin-positive	Isolated single cells	cCGH, Microsatellite analysis, PCR-RFLP, QPCR	Copy number, LOH
Watson et al, 2007 Clin Cancer Res	Breast	Non-metastatic	EPCAM-positive/Cytokerati n-positive	Enriched bone marrow	Expression microarray, QPCR	Gene Expression
Fuhrmann et al, 2008 Nucleic Acids Res	Breast	n.d.	Cytokeratin-positive	Isolated single cells	cCGH, aCGH, QPCR	Copy number
Stoecklein et al, 2008 Cancer Cell	Esophageal	Non-metastatic and metastatic	Cytokeratin-positive	Isolated single cells	cCGH, QPCR	Copy number
Holcomb et al, 2008 Cancer Res	Prostate	Non-metastatic and metastatic	EPCAM-positive	Pooled cells (10-20)	aCGH	Copy number
Weckermann et al, 2009 J Clin Oncol	Prostate	Non-metastatic and metastatic	Cytokeratin-positive	Isolated single cells	cCGH	Copy number
Mathiesen et al, 2012 Int J Cancer	Breast	Non-metastatic and metastatic	Cytokeratin-positive	Isolated single cells	aCGH	Copy number
Siddappa et al, 2012 Breast Cancer Res Treat	Breast	Non-metastatic	Expression of tumor-specific genes	Enriched bone marrow	Digital molecular barcoding, QPCR	Gene Expression
Moller et al, 2013 Front Oncol	Breast	Non-metastatic	Cytokeratin-positive	Isolated single cells	aCGH, Next generation sequencing	Copy number, Copy neutral LOH
Czyz et al, 2014 PLoS One	Breast	Metastatic	Cytokeratin-positive	Isolated single cells	aCGH	Copy number
Chery et al, 2014 Oncotarget	Prostate	Non-metastatic and metastatic	EPCAM-positive, CD45-negative	Isolated single cells	Expression microarray	Gene Expression
Guzvic et al, 2014 Cancer Res	Prostate	Non-metastatic and metastatic	EPCAM-positive	Isolated single cells	PCR, cCGH, aCGH	Gene expression, Copy number
Wu et al J Mol Diagn 2016	Prostate	Metastatic	EPCAM-positive, CD45-negative	Pooled cells (2-40)	SNP CGH arrays, nCounter	Copy number
Demeulemeester et al, 2016 Genome Biol	Breast	Non-metastatic	Cytokeratin-positive	Isolated single cells	Next generation sequencing	Copy number and Mutation profiling
Schumacher et al, 2017 British J Cancer	Esophageal	Non-metastatic	Cytokeratin-positive	Isolated single cells	cCGH	Copy number
This study	Breast	Non-metastatic	EPCAM-positive, CD45-negative	Pooled cells (~20)	aCGH, Multiplex QPCR	Gene expression, Copy number

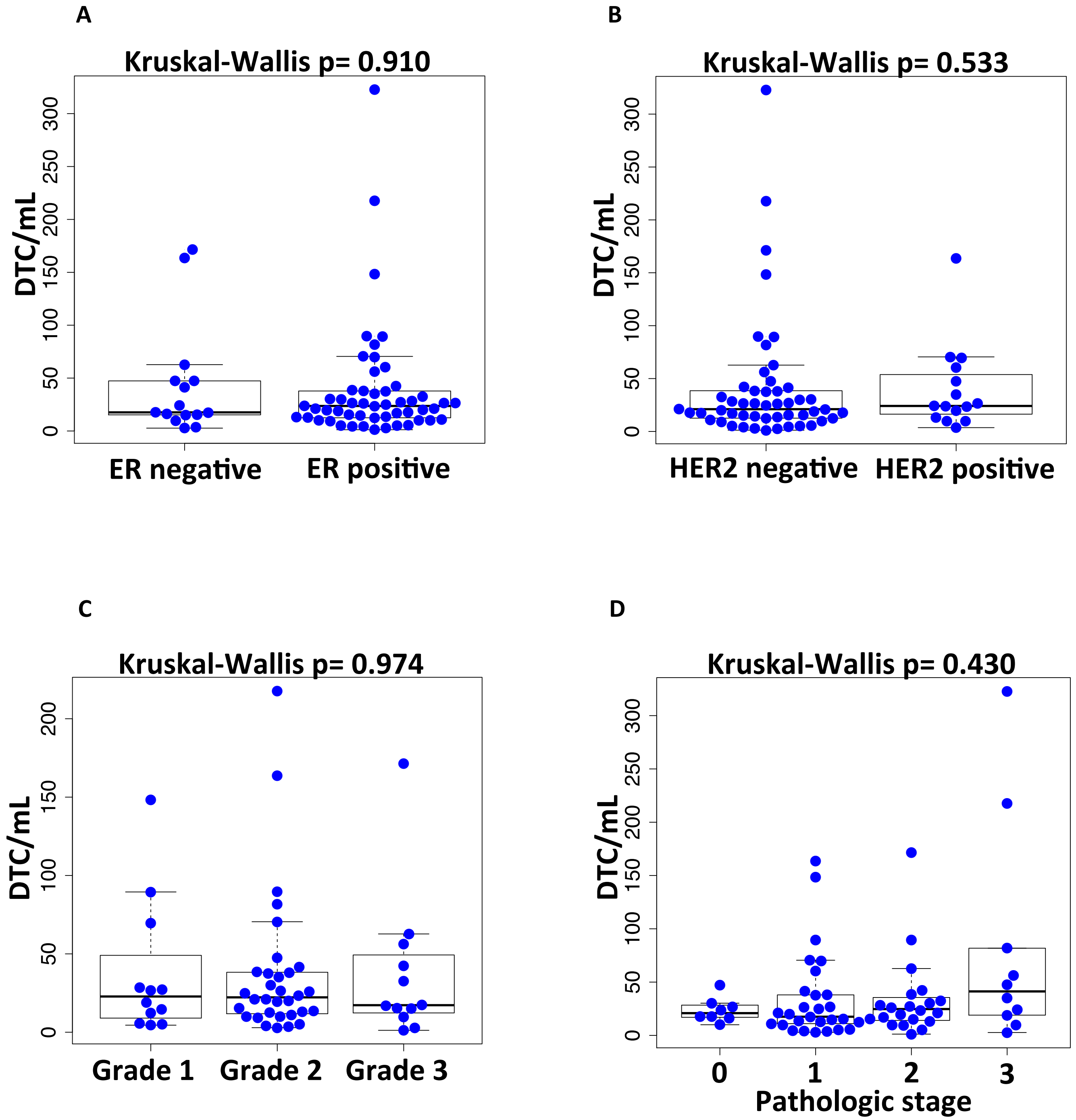

Supplementary Figure 1. Correlation between DTC concentration in bone marrow (DTC/mL) and patient clinical characteristics. A) ER status; B) HER2 status; C) Grade; D) pathological stage. Results for association between DTC/mL vs. lymph node status and treatment received are shown in **Figure 1** in the main text.

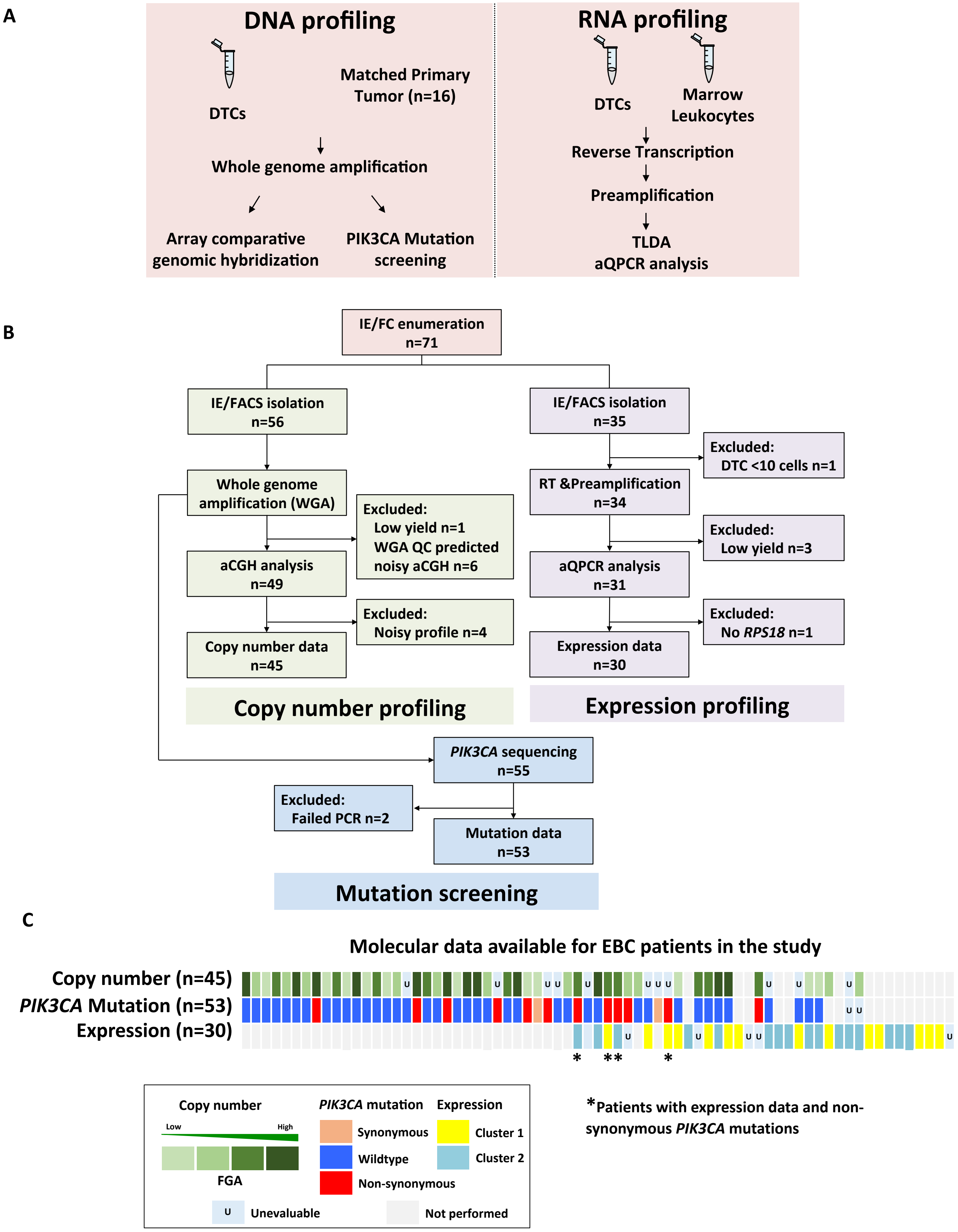

A
DNA profiling
DTCs
Matched Primary Tumor (n=16)
Whole genome amplification
Array comparative genomic hybridization
PIK3CA Mutation screening
RNA profiling
DTCs
Marrow Leukocytes
Reverse Transcription
Preamplification
TLDA aQPCR analysis
B
IE/FC enumeration n=71
IE/FACS isolation n=56
IE/FACS isolation n=35
Excluded: DTC <10 cells n=1
Whole genome amplification (WGA)
RT &Preamplification n=34
Excluded: Low yield n=1 WGA QC predicted noisy aCGH n=6
Excluded: Low yield n=3
aCGH analysis n=49
aQPCR analysis n=31
Excluded: Noisy profile n=4
Excluded: No RPS18 n=1
Copy number data n=45
Expression data n=30
Copy number profiling
Expression profiling
PIK3CA sequencing n=55
Excluded: Failed PCR n=2
Mutation data n=53
Mutation screening
C
Molecular data available for EBC patients in the study
Copy number (n=45)
PIK3CA Mutation (n=53)
Expression (n=30)
Copy number
Low
High
FGA
PIK3CA mutation
Synonymous
Wildtype
Non-synonymous
Expression
Cluster 1
Cluster 2
Unevaluable
Not performed
*Patients with expression data and non-synonymous PIK3CA mutations

Supplementary Figure 2. Study schema and workflow. A) Flow chart of sample processing for isolation, and DNA and RNA profiling of disseminated tumor cells (DTCs); B) Performance of each sample at each quality control (QC) step; C) Summary of the molecular data available for each of the 71 patients in the study.

A

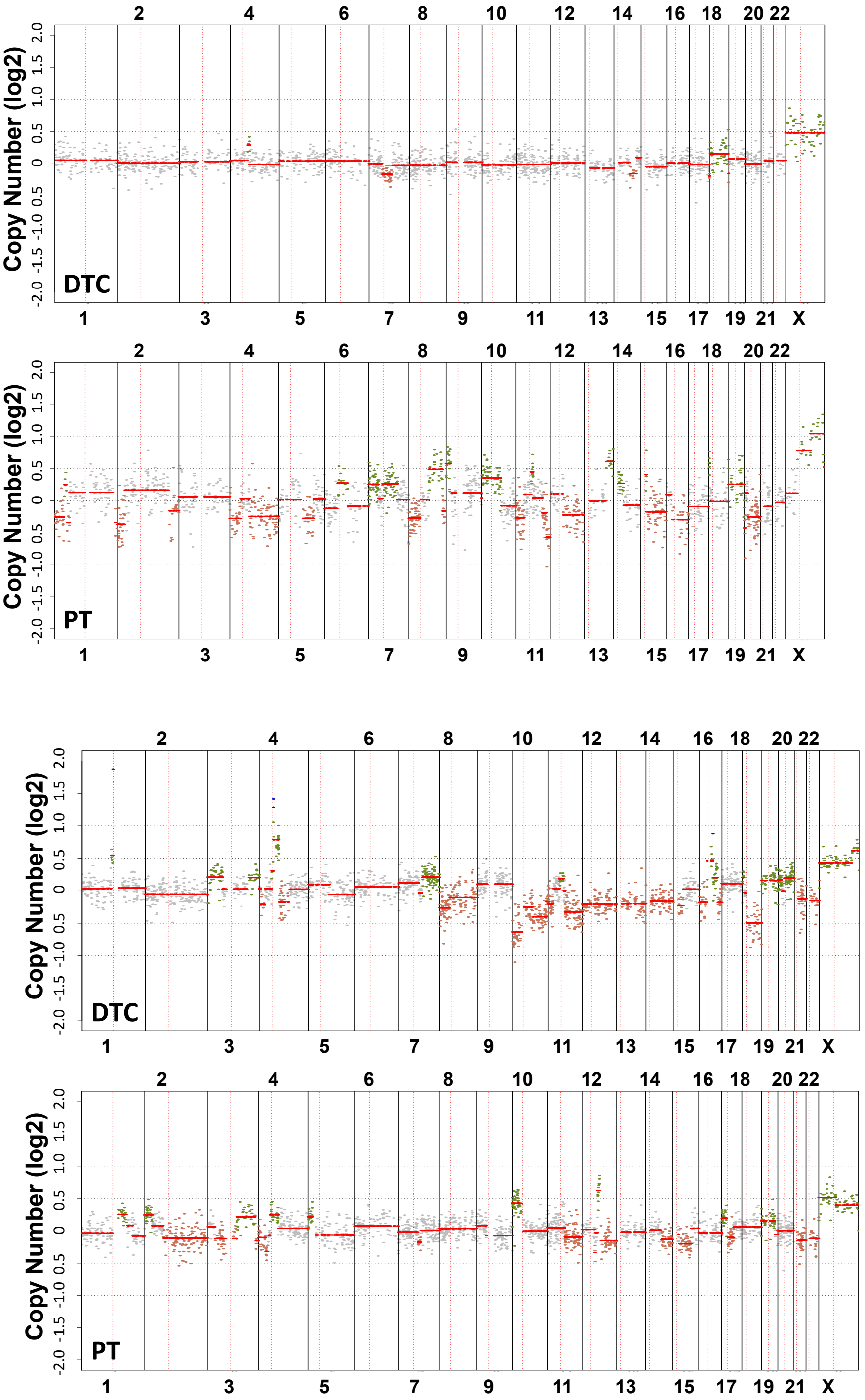
Copy Number (log2)
DTC
PT

B

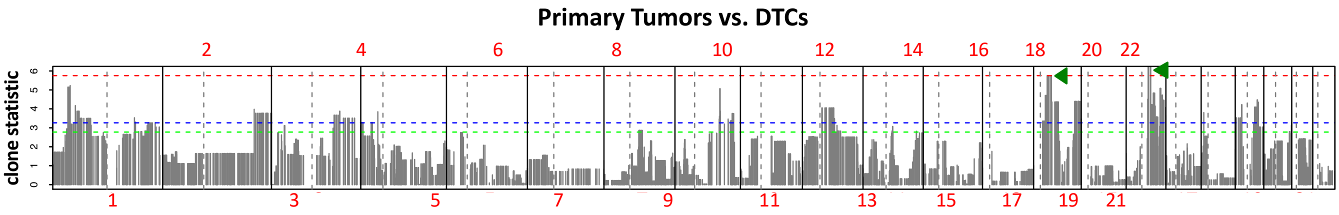
Primary Tumors vs. DTCs
clone statistic

Supplementary Figure 3. Copy number profiling of DTCs by bacterial artificial chromosome (BAC) array comparative genomic hybridization analysis. A) Representative genomic profiles from two patients. Copy number status (log2 ratio value) for each BAC clone is plotted on the y-axis. The x-axis represents the genomic position of each BAC clone on the array, with chromosome numbers indicated. Vertical solid lines indicate chromosome boundaries, and vertical red dashed line represents the centromeric region dividing each chromosome into the p- or short arm (to the left of centromere) and the q- or long arm (to the right of the centromere). Colors represent copy number status: green = gain, orange = loss, blue = amplification, red line = segmented value. B) For each BAC clone, a linear model was fit with the segmented value as the response variable and sample type as the predictor variable along with patient ID as a covariate using primary tumor and DTC samples only. Q-values were then computed to correct for multiple testing. Green, blue and red horizontal dotted lines mark the 0.1, 0.05, and 0.01 q-value cutoffs.

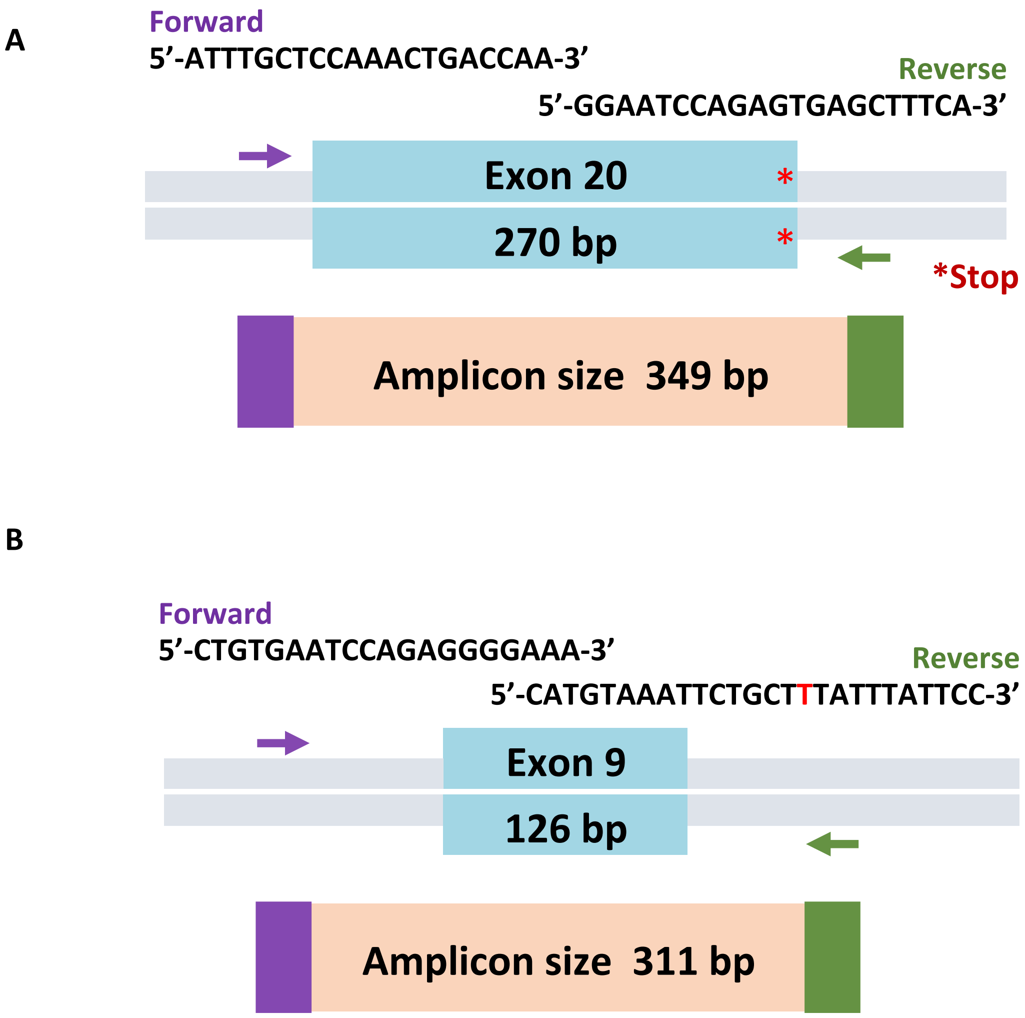

Supplementary Figure 4. *PIK3CA* mutation screening in DTCs by Sanger sequencing analysis. Primers for PCR amplification of complete (A) Exon 9 and (B) Exon 20, and expected amplicon sizes.

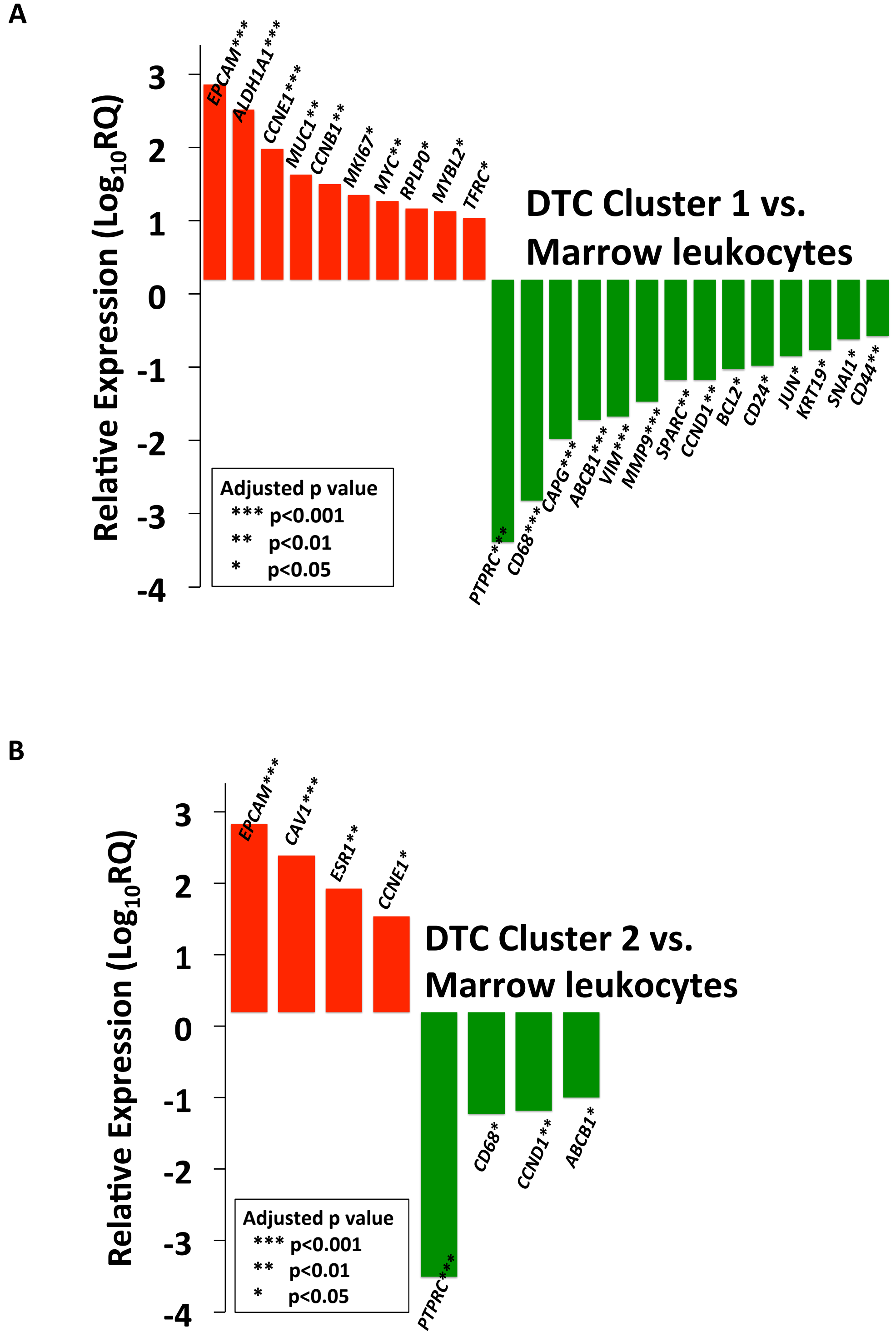

Supplementary Figure 5. Expression profiling of DTCs and marrow leukocytes by multiplexed aQPCR analysis. Differentially expressed genes between samples in (A) DTC clusters 1 and (B) DTC cluster 2 vs. marrow leukocytes. Red represents up-regulation and green represents down-regulation.

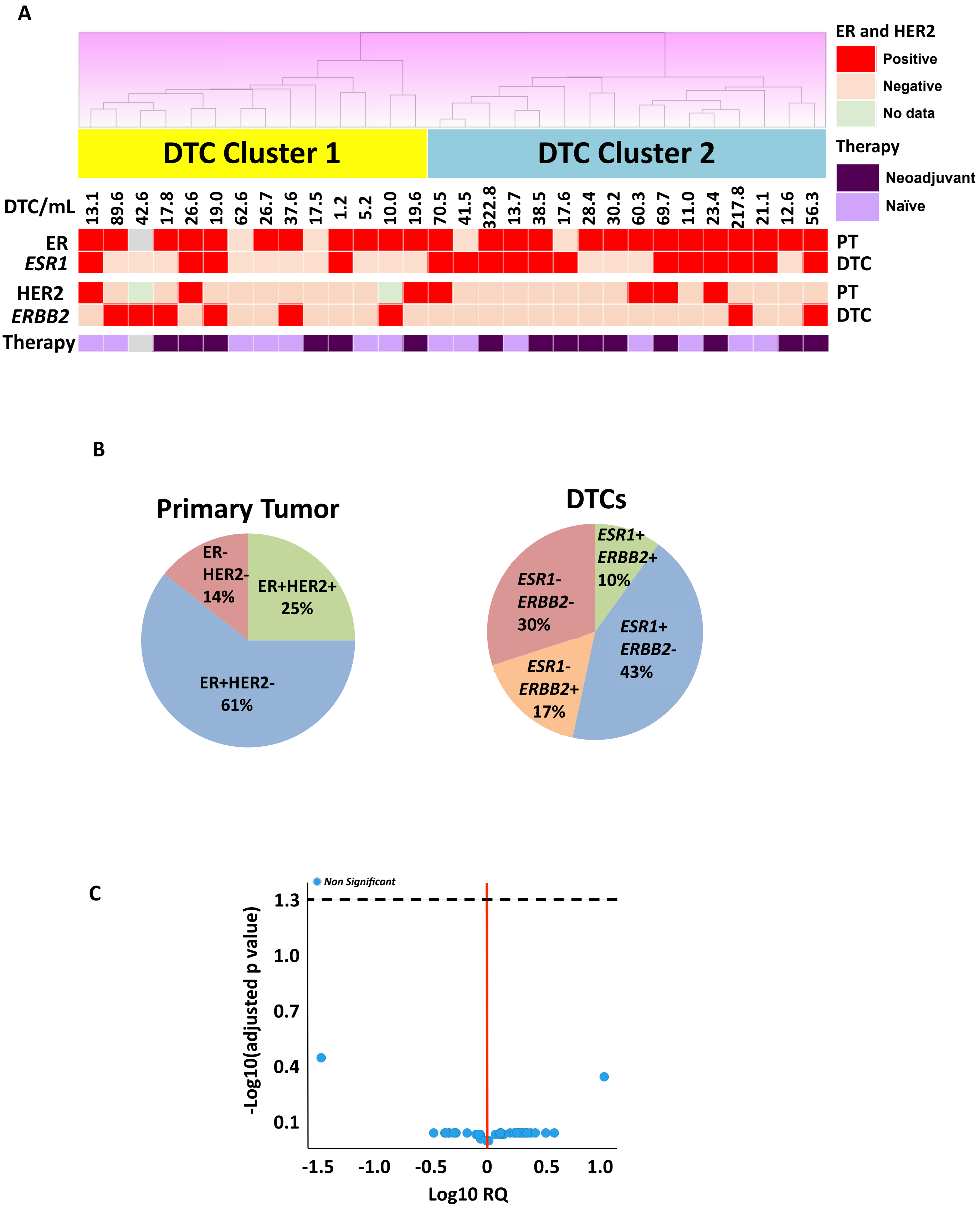
A
ER and HER2
Positive
Negative
No data
Therapy
Neoadjuvant
Naïve
DTC Cluster 1
DTC Cluster 2
DTC/mL
13.1
89.6
42.6
17.8
26.6
19.0
62.6
26.7
37.6
17.5
1.2
5.2
10.0
19.6
70.5
41.5
322.8
13.7
38.5
17.6
28.4
30.2
60.3
69.7
11.0
23.4
217.8
21.1
12.6
56.3
ER
ESR1
HER2
ERBB2
Therapy
PT
DTC
PT
DTC
B
Primary Tumor
ER-HER2-14%
ER+HER2+ 25%
ER+HER2- 61%
DTCs
ESR1+ ERBB2+ 10%
ESR1- ERBB2- 30%
ESR1+ ERBB2- 43%
ESR1- ERBB2+ 17%
C
Non Significant
-Log10(adjusted p value)
1.3
1.0
0.7
0.4
0.1
-1.5
-1.0
-0.5
0
0.5
1.0
Log10 RQ

Supplementary Figure 6. Expression phenotypes of DTCs. A) Unsupervised hierarchical clustering analysis of DTCs samples along with the concentration of DTCs (DTC per mL of bone marrow), *ESR1*/ER and *ERBB2*/HER2 status of DTCs and matched primary tumor, and treatment received. B) Proportion of receptor subtypes in DTCs and matched primary tumors; C) Volcano plot showing no significant genes differentially expressed between DTCs from patients who received neoadjuvant therapy vs. those show did not.

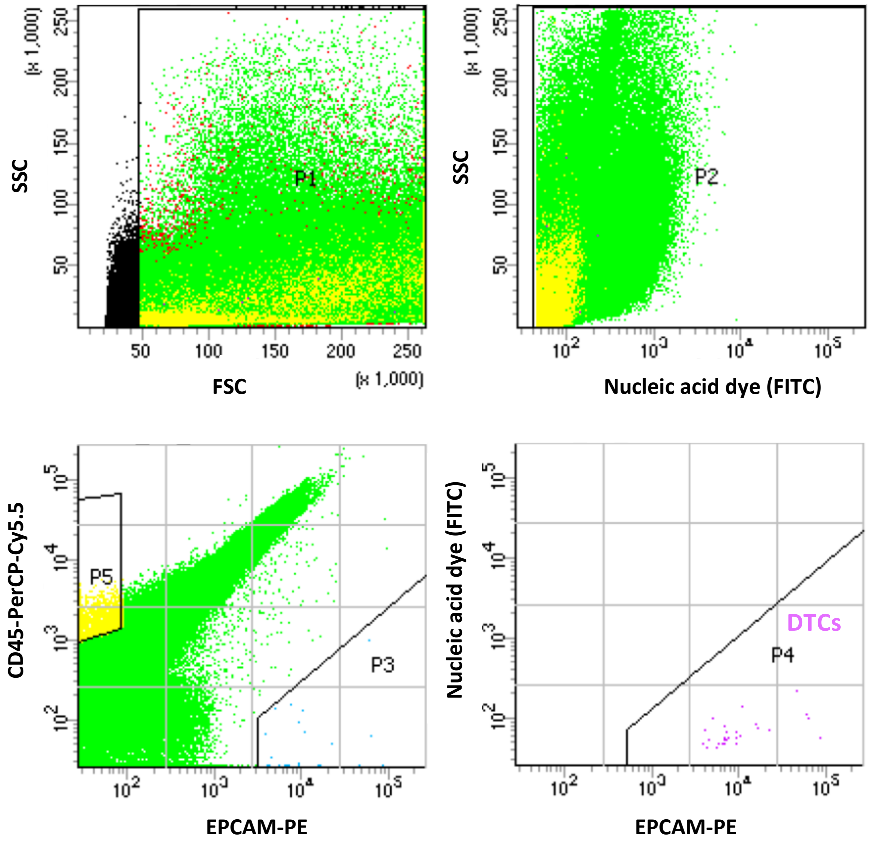

Population	#Events	%Parent	%Total
All Events	5,632,022	####	100.0
FSC Scaling	255,111	4.5	4.5
P1	4,876,773	86.6	86.6
P2	781,824	16.0	13.9
P3	27	0.0	0.0
P4 DTCs	27	100.0	0.0
P5	10,318	1.3	0.2

Supplementary Figure 7. A representative fluorescence-activated cell sorting (FACS) plot for detection and isolation of DTCs from bone marrow of a breast cancer patient. Bone marrow was incubated with two monoclonal antibodies (mAb) against EPCAM: (1) MJ-37 clone conjugated to magnetic beads and (2) EBA-1 clone) conjugated to phycoerythrin-labeled (EPCAM-PE). The mixture was subjected to a magnetic field to enrich for EPCAM-positive cells, and the enriched fraction was then analyzed by multiparameter FACS. The x and y axes for the different panels in the FACS plot shown are: (1) Top left panel—forward scatter (FSC)

vs. side scatter (SSC); (2) Top right panel—nucleic acid dye (Thioflavin detected in the FITC channel) vs. SSC; (3) Middle left panel—EPCAM PE vs. anti-CD45 (2D1 clone) conjugated to peridinin-chlorophyll-protein-Cy5.5 (CD45-PerCP-Cy5.5); (4) Middle right panel—EPCAM-PE vs. nucleic acid dye. Events in the P4 gate were considered DTCs, defined as EPCAM-positive, CD45-negative and nucleated; (5) Bottom panel—Number and percentages of events per population. Representative marrow leukocytes were collected in the P5 gate (CD45-positive, EPCAM-negative).
